# Radiosynthesis and In Vitro Evaluation of [^11^C]tozadenant as Adenosine A_2A_ Receptor Radioligand

**DOI:** 10.3390/molecules29051089

**Published:** 2024-02-29

**Authors:** Swen Humpert, Daniela Schneider, Markus Lang, Annette Schulze, Felix Neumaier, Marcus Holschbach, Dirk Bier, Bernd Neumaier

**Affiliations:** 1 Forschungszentrum Jülich GmbH, Institute of Neuroscience and Medicine, Nuclear Chemistry (INM-5), Wilhelm-Johnen-Str., 52428 Jülich, Germany; s.humpert@fz-juelich.de (S.H.); d.schneider@fz-juelich.de (D.S.); mar.lang@fz-juelich.de (M.L.); a.schulze@fz-juelich.de (A.S.); f.neumaier@fz-juelich.de (F.N.); m.holschbach@fz-juelich.de (M.H.); d.bier@fz-juelich.de (D.B.); 2 Institute of Radiochemistry and Experimental Molecular Imaging, Faculty of Medicine and University Hospital Cologne, University of Cologne, Kerpener Str. 62, 50937 Cologne, Germany

**Keywords:** adenosine A_2A_ receptor, carbon-11, positron emission tomography, tozadenant, radiolabeling

## Abstract

Tozadenant (4-hydroxy-*N*-(4-methoxy-7-morpholinobenzo[*d*]thiazol-2-yl)-4-methylpiperidine-1-carboxamide) is a highly selective adenosine A_2A_ receptor (A_2A_R) antagonist and a promising lead structure for the development of A_2A_R-selective positron emission tomography (PET) probes. Although several ^18^F-labelled tozadenant derivatives showed favorable in vitro properties, recent in vivo PET studies observed poor brain penetration and lower specific binding than anticipated from the in vitro data. While these findings might be attributable to the structural modification associated with ^18^F-labelling, they could also reflect inherent properties of the parent compound. However, PET studies with radioisotopologues of tozadenant to evaluate its cerebral pharmacokinetics and brain distribution are still lacking. In the present work, we applied *N*-Boc-*O*-desmethyltozadenant as a suitable precursor for the preparation of [*O*-methyl-^11^C]tozadenant ([^11^C]tozadenant) by *O*-methylation with [^11^C]methyl iodide followed by acidic deprotection. This approach afforded [^11^C]tozadenant in radiochemical yields of 18 ± 2%, with molar activities of 50–60 GBq/µmol (1300–1600 mCi/µmol) and radiochemical purities of 95 ± 3%. In addition, in vitro autoradiography in pig and rat brain slices demonstrated the expected striatal accumulation pattern and confirmed the A_2A_R specificity of the radioligand, making it a promising tool for in vivo PET studies on the cerebral pharmacokinetics and brain distribution of tozadenant.

## 1. Introduction

**Figure 1:**
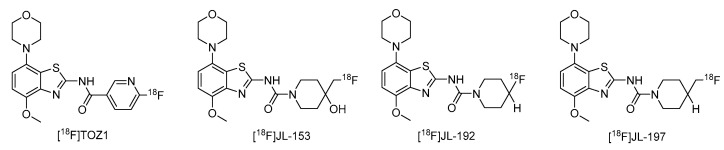
Tozadenant-derived ^18^F-labeled A_2A_R antagonists for PET imaging [14,22].

The nucleoside adenosine is a pleiotropic signaling molecule that mediates its cellular effects by activation of four different adenosine receptor (AR) subtypes (A_1_R, A_2A_R, A_2B_R and A_3_R) [1]. The predominant AR subtypes in the central nervous system are A_1_R and A_2A_R, which participate in the regulation of many physiological processes like wakefulness, cognition and motor function [2,3,4]. While A_1_Rs are widely expressed throughout the whole brain, A_2A_Rs show a more restricted expression pattern, with high levels in the striatum and much lower levels in extra-striatal brain regions [1,5,6,7,8]. In addition, many neuroinflammatory and neurodegenerative conditions have been shown to be associated with changes in the distribution and/or function of A_2A_Rs, making them attractive therapeutic targets for a wide spectrum of neurological diseases [8,9,10]. For example, inhibition of the striatopallidal motor pathway with selective A_2A_R antagonists like tozadenant (4-hydroxy-*N*-(4-methoxy-7-morpholin-4-yl-1,3-benzo[*d*]thiazol-2-yl)-4-methylpiperidine-1-carboxamide) has been pursued as a non-dopaminergic treatment option for Parkinson’s disease (PD) [11,12]. Although tozadenant effectively improved PD symptomatology with virtually no off-target effects, reports of hematological toxicity during long-term treatment necessitated early termination of phase 3 clinical trials [13]. Nevertheless, its high affinity (K_i_ = 3.9–4.9 nM for human A_2A_R) [8,14], unique target selectivity [15] and high in vivo stability [16,17] position tozadenant as a promising lead structure for the development of positron emission tomography (PET) probes, which could be used to visualize and quantify cerebral A_2A_Rs in patients with PD or other neurological diseases [9,18]. Thus, taking into account reported densities of A_2A_Rs in the human striatum (B_max_ = 259–870 fmol/mg protein [8,19,20]), the estimated (in vitro) binding potential (B_max_/K_i_) for tozadenant (>5) indicates that radiolabeled analogs with comparable or superior affinity could be suitable for quantitative comparisons with PET imaging [21]. Accordingly, a number of ^18^F-labeled tozadenant derivates have been developed (Figure 1) and evaluated by in vitro autoradiography, which generally demonstrated an excellent A_2A_R specificity of these radioligands [14,22]. On the other hand, preclinical in vivo evaluation of ^18^F-labeled tozadenant derivates performed so far revealed a rather low brain penetration and considerably lower specific binding than anticipated from in vitro studies [22,23].

Given that the cerebral pharmacokinetics and brain distribution of tozadenant itself have never been examined, these findings could reflect undesirable in vivo properties of the benzothiazole scaffold and/or negative effects of the structural modifications associated with ^18^F-labeling. For example, while the quotient of receptor density and affinity (i.e., B_max_/K_i_) provides an upper limit for striatal accumulation, slow blood–brain barrier (BBB) penetration, active export across the BBB or other pharmacokinetic features of the benzothiazole scaffold itself (e.g., unsuitable lipophilicity) could significantly reduce the in vivo availability of tozadenant or derived radioligands in the brain [8]. Thus, although existing evidence indicates that the extent of brain entry during continued treatment with tozadenant was sufficient for clinical efficiency, neither the kinetics of brain penetration nor the lipophilicity or interaction with drug excretion pumps like P-gp have been studied to date and multiple probabilistic scores indicative of BBB penetration were indeed rather low [24]. Alternatively, ^18^F-labeling might have reduced BBB penetration or resulted in other pharmacokinetic changes that negatively influence in vivo performance, in which case derivatives with suitable imaging properties could potentially be obtained through the use of alternative radiolabeling strategies. However, so far, isotopologues of tozadenant labeled with carbon-11 which could be used to distinguish between these two possibilities have not been disclosed. In the present article, we describe a protocol for automated production of the ^11^C-labeled tozadenant isotopologue [*O*-methyl-^11^C]tozadenant ([^11^C]tozadenant) and a preliminary in vitro evaluation of this novel radioligand.

## 2. Results and Discussion

### 2.1. ^11^C-Methylation of Unprotected Desmethyl Precursors

**Figure 2:**
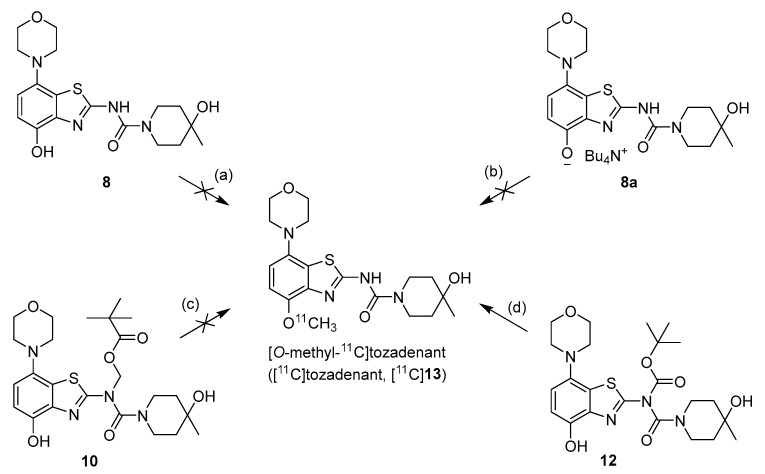
Investigated radiolabeling protocols for the preparation of [^11^C]tozadenant ([^11^C]**13**). Conditions: (a) [^11^C]MeI, base, solvent, t; (b) [^11^C]MeOTf, acetone or MeCN, rt; (c) [^11^C]MeI, 1 m NaOH_aq_ (2 equiv), DMF, 60 °C; (d) [^11^C]MeI, 1 m NaOH_aq_ (2 equiv), solvent, 60 °C.

We initially selected *O*-methylation of *O*-desmethyltozadenant (**8**) with [^11^C]MeI (Figure 2, route a) as a straightforward route for the preparation of [^11^C]tozadenant ([^11^C]**13**).

**Figure 3:**
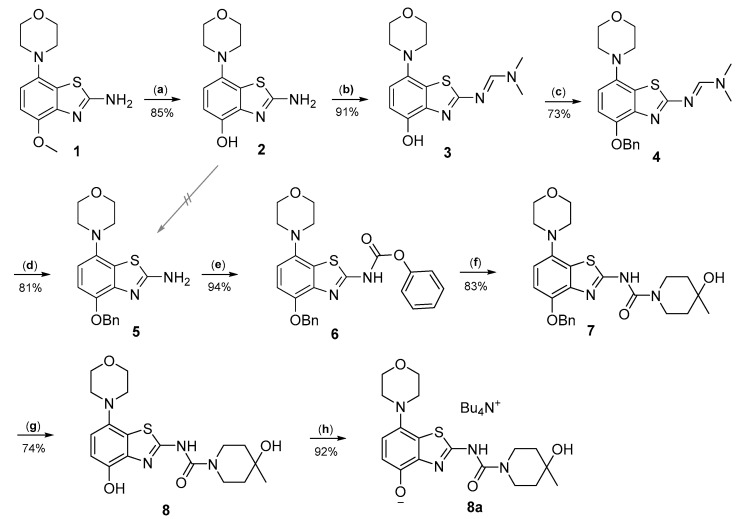
Preparation of radiolabeling precursors **8** and **8a**. Conditions: (a) HBr_aq_, reflux; (b) DMF-DMA, DMF; (c) BnBr, Cs_2_CO_3_, DMF; (d) KOH, MeOH, reflux; (e) phenyl chloroformate, THF; (f) 4-methyl-4-piperidinol, DMSO, 50 °C; (g) Pd/C, ammonium formate, DMF, 50 °C; (h) Bu_4_NOH, MeOH.

Radiomethylation precursor **8** was prepared in seven steps, starting from known 4-methoxy-7-morpholinobenzo[*d*]thiazol-2-amine (**1**) [14] (Figure 3). To this end, **1** was first demethylated with boiling aqueous 48% hydrobromic acid, which afforded 2-amino-7-morpholinobenzo[*d*]thiazol-4-ol (**2**) in excellent yield. Although subsequent Cs_2_CO_3_-supported *O*-alkylation of **2** with benzyl bromide directly furnished benzyl ether **5**, the desired product was obtained in low yields and always contaminated with a lipophilic impurity that proved difficult to remove by chromatography. In addition, sufficient chemoselectivity for benzylation of the phenolic *OH* over the amino group in **2** could be achieved neither at low temperatures nor by the use of stoichiometric reagent amounts, which necessitated chromatographic purification and resulted in low yields of the desired benzyl ether. As such, we instead opted to perform *O*-benzylation after appropriate protection of the amino group. Condensation of **2** with benzaldehyde to form the imine of the primary exocyclic amine while preserving the phenolic *OH* was unsuccessful. As an alternative, the amino group was protected by reaction of **2** with *N*,*N*-dimethylformamide dimethyl acetal (DMF-DMA), which furnished the corresponding *N*,*N*-dimethylformamidine **3** in an over 90% yield. Subsequent Cs_2_CO_3_-supported *O*-benzylation of **3** at ambient temperature followed by *N*-deprotection of the resulting intermediate **4** with potassium hydroxide afforded benzyl ether **5** in a 77% yield over two steps. Compound **5** was then condensed with phenyl chloroformate using pyridine as a base to prepare phenyl carbamate **6** as a stable solid in almost quantitative yield. Next, the asymmetric *N*,*N*′-disubstituted urea **7** was synthesized by aminolysis of **6** under neutral conditions with DMSO as reaction solvent [25]. To this end, **6** was treated with a slight excess of 4-hydroxy-4-methylpiperidine at 50 °C, which rapidly generated **7** in an 83% yield. Finally, **7** was *O*-debenzylated by catalytic transfer hydrogenation (CTH) with Pd/C and a sixfold excess of ammonium formate as hydrogen donor [26] for ten min, which afforded precursor **8** in 74% yield.

Unexpectedly, radiomethylation of **8** with [^11^C]MeI using various reaction solvents, bases and reaction conditions failed and only unidentified products could be detected. In an attempt to enhance the nucleophilicity of the phenolic oxygen and the chemoselectivity of the methylation reaction [27], we also prepared the tetrabutylammonium (TBA) salt of precursor **8**. To this end, a suspension of **8** (1.2 equivalents) in MeOH was reacted with TBA hydroxide (1 equivalent) in MeOH followed by separation of unreacted precursor **8** by centrifugation, which afforded **8a** in 90% yield.

**Figure 4:**
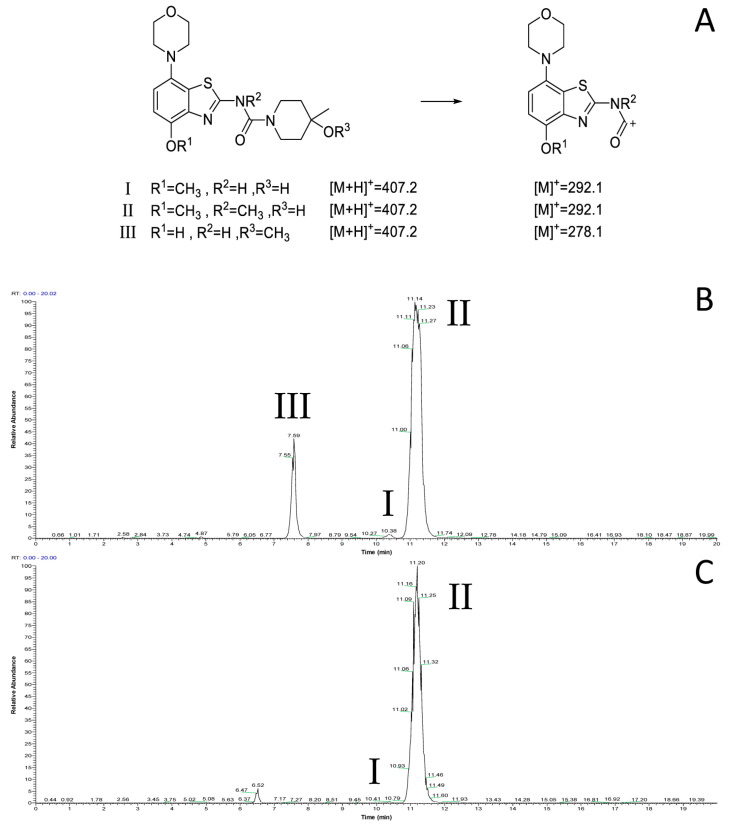
ESI(+) HPLC-MS analysis of the crude product solution obtained by reaction of precursor **8** with methyl triflate. (**A**) Calculated *m*/*z* values for the different main products and the derived benzothiazol fragments. (**B**) Total ion current (TIC) chromatogram filtered to *m*/*z* 407, which corresponds to tozadenant (t_R_ = 10.4 min) and its regioisomers (t_R_ = 7.6 and 11.2 min). (**C**) TIC filtered to *m*/*z* 292, which corresponds to the methylated benzothiazol fragment expected for tozadenant and its *N*-methylated regioisomer. The peak at 6.5 min (*m*/*z* = 421) corresponds to a product formed by double methylation and is therefore not relevant for no-carrier-added ^11^C-methylation reactions. HPLC conditions: Column: Phenomenex Kinetex® F5, 5 µm, 250 × 4.6 mm; eluent: 25% MeCN in H_2_O + 0.2% HCOOH, flow rate: 1.5 mL/min.

However, radiomethylation of **8a** with [^11^C]methyl trifluoromethanesulfonate ([^11^C]methyl triflate) [28] in acetone or acetonitrile (Figure 2, route b) did not yield the desired product. To identify possible causes, we performed methylation experiments with sub-stoichiometric amounts of non-radioactive methyl triflate in acetone and analyzed the reaction mixture by ESI(+) HPLC-MS. Besides small amounts of several unidentified polar side products, these experiments revealed the formation of three main products with the *m*/*z* of tozadenant (Figure 4). Comparison of their retention times with that of the reference compound demonstrated that tozadenant was the least abundant product (I in Figure 4) and obtained in less than one percent yield (determined by comparison of the integrated UV absorption signals of the three compounds). ESI in-source fragmentation of the less polar main product (II in Figure 4), which constituted approximately 90%, resulted in formation of a fragment characteristic for the methylated aminobenzothiazol moiety (*m*/*z* 293) [17], suggesting that it corresponded to the *N*-methylated regioisomer of tozadenant. The slightly more polar third product (III in Figure 4) could not be fragmented and most likely resulted from methylation of the tertiary hydroxy group as the only remaining nucleophile that would not give rise to a highly polar onium product. Interestingly, this product constituted approximately 10% and was thus formed in more than 10-fold higher amounts than tozadenant, indicating a surprisingly low nucleophilicity of the phenolic hydroxy group. These results, although unexpected, were in line with the competing *O*- and *N*-benzylation reactions observed during precursor preparation.

### 2.2. ^11^C-Methylation of a Base-Labile Protected Desmethyl Precursor

**Figure 5:**
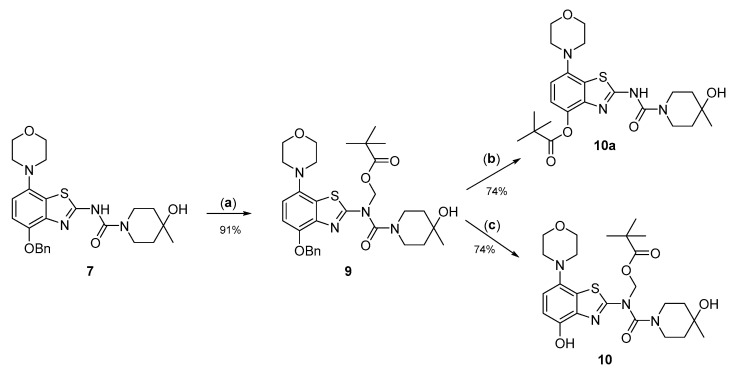
Preparation of *N*-Pom-protected precursor **10**. Depending on reaction conditions, *O*-debenzylation of **9** by catalytic transfer hydrogenation furnished either **10** or (due to base-induced rearrangement) **10a**. Conditions: (a) Pom-Cl, K_2_CO_3_, DMF, 60 °C; (b) Pd/C, ammonium formate, DMF, 55 °C; (c) Pd/C, ammonium formate, MeOH-AcOH, 55 °C.

Due to its relatively high nucleophilicity, we next assessed whether radiomethylation could be achieved after protection of the amidic nitrogen in precursor **8** with a pivaloyloxymethyl (Pom) protecting group (Figure 2, route c). Accordingly, *O*-benzyl urea **7** was reacted with chloromethyl pivalate and potassium carbonate in DMF to produce *N*-protected intermediate **9** in a >90% yield (Figure 5). Subsequent *O*-debenzylation of **9** by CTH under the same conditions as described above for **7** resulted in formation of pivaloyl ester **10a** instead of the expected Pom-protected radiolabeling precursor **10**. This result can most likely be explained by rapid base-induced migration of the Pom protecting group with the concomitant release of formaldehyde, as previously observed during Pom-protection of the nucleobase adenine [29]. Thus, catalytic decomposition of ammonium formate is well known to release ammonia [30], which could result in sufficient alkalinity to induce the observed acyl migration. In line with this assumption, *O*-debenzylation of **9** by CTH in a mixture of MeOH and acetic acid instead of DMF furnished the desired radiolabeling precursor **10** in a 74% yield as a bench-stable powder.

**Figure 6:**
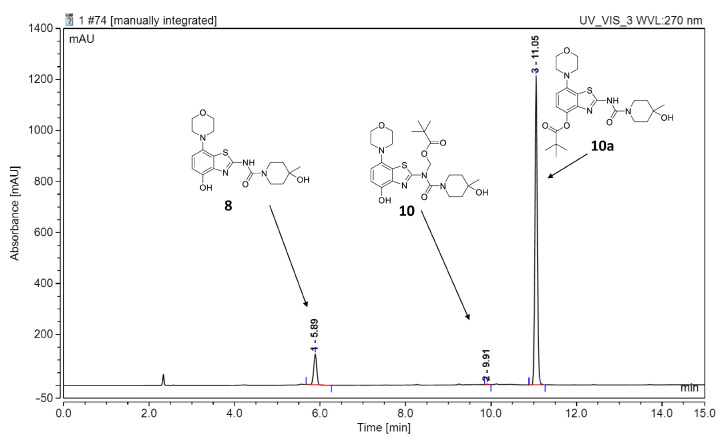
ESI HPLC-MS analysis of the products formed upon treatment of precursor **10** in DMF with two equivalents of NaOH and heating to 60 °C to simulate radiolabeling conditions. HPLC conditions: Column: Phenomenex Kinetex® F5 5 µm, 250 × 4.6 mm; eluent (solvent A = 0.2% HCOOH in H_2_O, solvent B = 0.2% HCOOH in MeCN): linear gradient: 0 min 20% B, 15 min: 80% B; flow rate: 1.5 mL/min.

However, first screening experiments by methylation of **10** with non-radioactive MeI (0.1 equivalents) using different solvents (DMF, DMSO or acetonitrile), bases (Cs_2_CO_3_, K_2_CO_3_, NaOH, (*n*-Bu)_4_NOH or NaH) and reaction temperatures (20–120 °C) followed by deprotection also did not furnish the desired product tozadenant. Suspecting insufficient base stability of the precursor, a solution of **10** in DMF was treated with 1 N aqueous NaOH (two equivalents) and heated to 60 °C to simulate the slightly basic radiolabeling conditions. ESI HPLC-MS analysis revealed almost complete disappearance of **10** (*m*/*z* 507) within minutes and formation of two new compounds (*m*/*z* 477 and *m*/*z* 393) in a ratio of 9:1 (Figure 6). Based on the mass difference of 30 units compared to **10**, the main product (*m*/*z* 477) was formed by elimination of formaldehyde, indicating that it corresponded to compound **10a** produced by base-induced acyl migration. The mass of the second product (*m*/*z* 393) was consistent with that of the Pom-deprotected product, indicating that it corresponded to compound **8**. Thus, precursor **10** proved to be unsuitable for preparation of [^11^C]tozadenant, since even the slightly basic conditions required for radiomethylation resulted in rapid base-induced acyl migration with formation of pivaloyl ester **10a** and (to a lesser extent) hydrolysis into the non-protected precursor **8**.

### 2.3. ^11^C-Methylation of an Acid-Labile Protected Desmethyl Precursor

**Figure 7:**
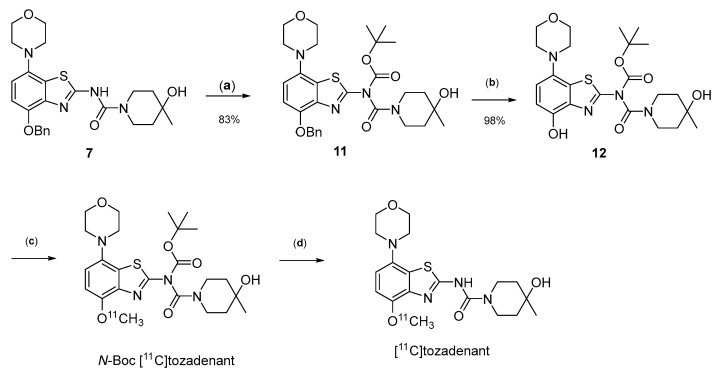
Preparation of [^11^C]tozadenant from the *N*-Boc protected precursor **12**. Conditions: (a) (Boc)_2_O, TEA, DMAP, THF, rt; (b) ammonium formate, Pd/C, DMF, 55 °C; (c) n.c.a. [^11^C]methyl iodide, NaOH, DMF, 60 °C; (d) conc. HCl, 100 °C.

Owing to its low stability, we next replaced the base-labile Pom-protecting group with a base-stable but acid-labile *tert*-butyloxycarbonyl (Boc) residue. To this end, *O*-benzyl urea **7** was reacted with di-*tert*-butyldicarbonate and 4-dimethylaminopyridine (DMAP)/triethylamine in THF, which afforded the *O*-Bn-*N*-Boc diprotected intermediate **11** in a yield of 89%. Subsequent *O*-debenzylation of **11** by CTH under the same conditions as described for **7** furnished the desired *N*-Boc protected radiomethylation precursor **12** as bench-stable crystals in near-quantitative yield (Figure 7).

**Figure 8:**
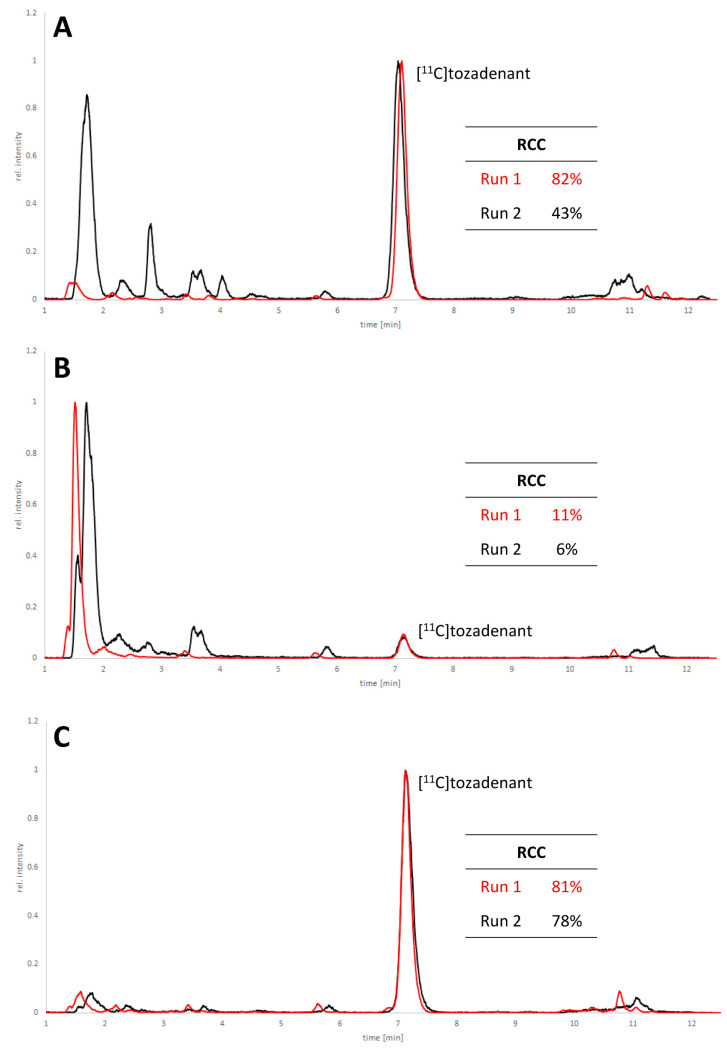
HPLC chromatograms of crude reaction mixtures obtained by ^11^C-methylation of precursor **12** with [^11^C]MeI in DMF (**A**), DMSO (**B**) or DMA (**C**) followed by de-protection of the radiolabeled intermediate. Two reactions were performed in close succession with each solvent (indicated in red and black) and all signals were normalized to the highest peak in the corresponding chromatogram. Reaction conditions: (i) Radiomethylation: precursor (2 µmol), 1 N NaOH_aq_ (4 µL), solvent (300 µL), 5 min, 60 °C; (ii) Deprotection: 37% HCl (150 µL), 5 min, 100 °C. HPLC conditions: Column: MultoKrom® 100-5 C18 250 × 4.6 mm; eluent: 0–10 min: 32.5% MeCN_aq_ + 0.2% HCOOH, 10–15 min: 80% MeCN_aq_ + 0.2% HCOOH; flow rate: 1.5 mL/min.

Non-radioactive screening experiments similar to those performed with **10** (i.e., treatment of precursor in DMF with 1 N aqueous NaOH and heating to 60 °C) indicated a sufficient base stability of **12**. Accordingly, radiomethylation of **12** was performed with [^11^C]MeI in DMF and 1 N aqueous NaOH (2 equivalents) for 5 min at 60 °C (Figure 2, Route d). Subsequent Boc-deprotection with concentrated aqueous HCl at 100 °C for 5 min furnished the desired tracer [^11^C]tozadenant, albeit in highly variable yields. Likewise, small-scale manual radiolabeling experiments performed in close succession produced distinctly different results, with a moderate radiochemical conversion (RCC) of 43% and large quantities of polar side products in one case and an excellent RCC of 82% and few side products in the other (Figure 8A). These discrepancies could potentially be explained by hydroxide-assisted thermal decomposition of DMF with formation of dimethylamine as a competing nucleophile in amounts that depended on the exact timing [31]. With this in mind, additional small-scale experiments were performed with DMSO or DMA as alternative reaction solvents. While the use of DMSO proved to be unsuitable and resulted in RCCs of only 5–10% (Figure 8B), ^11^C-methylation of **12** in DMA proceeded smoothly and afforded the desired tracer in reproducible RCCs of 78–81% with only a few minor side products (Figure 8C).

Having established suitable reaction parameters, [^11^C]tozadenant could be reliably produced on a custom-built automated synthesis module. After rapid semipreparative HPLC purification (Figure S1) and formulation, the tracer was obtained in radiochemical yields of 18 ± 2% (850–1250 MBq), radiochemical purities of 95 ± 3%, high chemical purities (Figure S2) and molar activities of 50–60 GBq/µmol (1300–1600 mCi/µmol) in an overall synthesis time of 40 min. All quality control parameters were within the specifications for preclinical in vivo studies (Table 1, n = 3).

### 2.4. Preliminary In Vitro Evaluation of [^11^C]tozadenant

**Figure 9:**
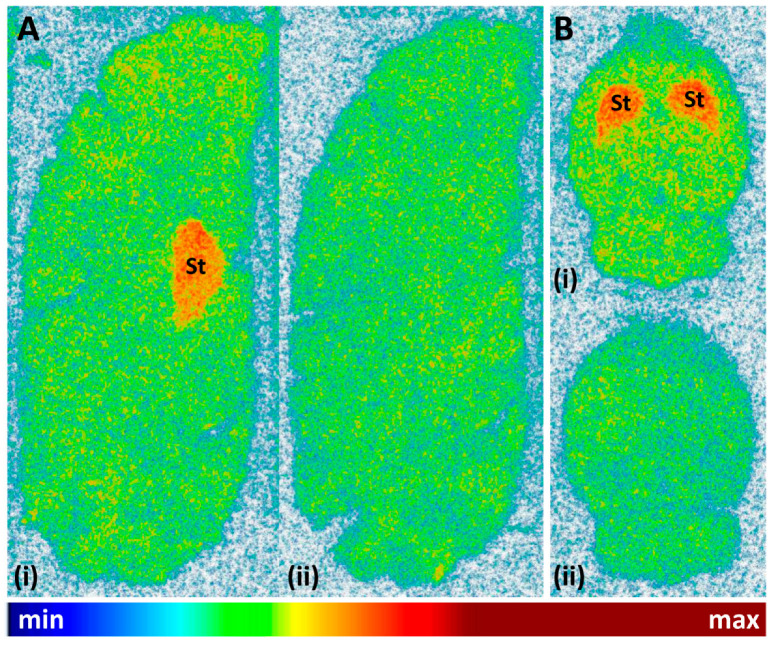
Representative autoradiographic images obtained after incubation of (**A**) sagittal pig brain slices or (**B**) horizontal rat brain slices with 12 kBq/mL (0.24 nM) [^11^C]tozadenant in the absence (i = total binding) or presence (ii = non-specific binding) of 1 µM ZM241385. Abbreviations: St = striatum.

Finally, the cerebral binding pattern of [^11^C]tozadenant was determined using in vitro autoradiography with brain slices from rat and pig as two common preclinical species. As illustrated in Figure 9, the regional distribution of radioactivity observed in these experiments was consistent with the established expression patterns of A_2A_Rs in both species [5,6,32], with pronounced accumulation in the striatum and much lower levels in extra-striatal brain regions. Specific binding of the radioligand, determined from the difference between total binding and binding in the presence of the A_2A_R-selective antagonist ZM241385 (1 µm), amounted to approximately 68%.

## 3. Materials and Methods

### 3.1. General Information

Unless noted otherwise, all reactions were carried out under argon at ambient temperature (22 ± 2°C) in oven-dried glassware. Standard inert atmosphere techniques were used in handling all air and moisture-sensitive reagents. Melting points were measured on a FP62—Melting Point Meter (Mettler Toledo, Gießen, Germany) and are uncorrected.

#### 3.1.1. Solvents and Reagents

Solvents were either purchased in sufficient purity (MerckKGaA, Darmstadt, Germany) or purified and dried using standard methods [33]. Chemicals were purchased from Merck (Taufkirchen, Germany), Activate Scientific (Prien, Germany) and ABCR (Karlsruhe, Germany) or prepared as described in the text. 2-Amino-4-methoxy-7-morpholinobenzothiazole (**1**), 2-amino-4-hydroxy-7-morpholinobenzothiazole (**2**) and the reference compound tozadenant (**13**) were prepared as described previously [14].

#### 3.1.2. Spectroscopy

Nuclear magnetic resonance (NMR) spectra were recorded in a 5% solution at 298 K (^1^H: 400.13 MHz; ^13^C: 100.61 MHz; ^19^F: 376.49 MHz) using a Bruker Avance Neo 400 (Bruker Bio Spin GmbH, Rheinstetten, Germany). The measured chemical shifts (δ) are reported in parts per million (ppm) relative to the residual solvent signals (for CDCl_3_, δ_H_ = 7.26, δ_C_ = 77.16; for DMSO-*d_6_*, δ_H_ = 2.50, δ_C_ = 50.32). The ^1^H-NMR spectra are reported as follows: δ, multiplicity, coupling constant *J* (in Hz) where appropriate, number of protons, assignment. The following abbreviations and their combinations are used when reporting NMR data: s = singlet, d = doublet, t = triplet, q = quartet, m = multiplet, s_br_ = broad singlet, m_obsc_ = obscured multiplet and v = very. NMR signals were assigned based on information from additional two-dimensional experiments (COSY, gHSQC, gHMBC, NOESY). Coupling constants for protons are given in the form of *^n^J*(^1^H, X), and those for carbons as *^n^J*(^13^C, ^19^F). All ^13^C- and ^19^F-NMR spectra were recorded under ^1^H-broadband decoupling (CPD). Compound names were generated by ChemBioDraw™ (CambridgeSoft) following IUPAC nomenclature. The numbering of the benzo[*d*]thiazol-2-amine (2-aminobenzothiazole) scaffold was performed as shown below.


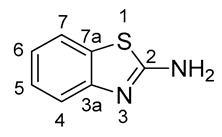


Low-resolution mass spectra were obtained in electrospray ionization (ESI-positive) mode with a Thermo Fischer Scientific MSQ Plus mass spectrometer (Thermo Fisher Scientific GmbH, Dreieich, Germany). The analytes were dissolved in MeOH (about 1 mg/mL) and injected directly through a valve on the ionisation interface. The flow rate of the eluent (MeOH/water/AcOH, 50/50/0.2, *v*/*v*/*v*) was 200 μL/min. Reported are the *m*/*z*-values of the pseudo-ion [M + H]^+^.

High-resolution mass spectra were determined in the Central Division of Analytical Chemistry at the Forschungszentrum Jülich. Elemental analyses were performed using HEKAtech GmbH (Wegberg, Germany). Analyses indicated by the symbols of the elements are within ±0.4% of the theoretical values.

#### 3.1.3. Chromatography

Thin-layer chromatography (TLC) on silica-coated aluminium sheets with a fluorescent indicator (ALUGRAM SIL G/UV_254_ Macherey-Nagel GmbH, Düren, Germany) was performed to monitor the progress of all reactions and to confirm the purity of the resulting products. The respective eluent was selected so that the R_f_ values of the individual compounds ranged from 0.2 to 0.8. Compounds were visualized under UV light and/or by iodine staining.

Flash chromatography was performed with a Büchi Pure C-815 flash chromatography system (BÜCHI Labortechnik AG, Flawil, Switzerland) equipped with multisignal (UV/ELSD) detection and FlashPure silica cartridges (particle size 40 µm) as a stationary phase. Solvent proportions are indicated in a volume/volume ratio.

### 3.2. Chemical Syntheses

#### 3.2.1. Preparation of 2-Amino-7-morpholinobenzo[*d*]thiazol-4-ol (**2**)


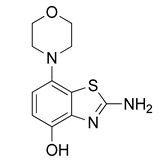


A solution of 2-amino-4-methoxy-7-morpholinobenzothiazole **1** (1.3 g, 5 mmol) in 48% HBr (8.9 m, 50 mL, 445 mmol, good quality: colorless to slightly yellowish) was stirred at 130 °C for 24 h. The resulting suspension was cooled to ambient temperature and left to stand overnight at 5 °C. The grey–brown solid was then collected by filtration and taken up into water (50–60 mL). Under efficient stirring, the mixture was titrated with 10 N aqueous sodium hydroxide until a precipitate started to form, followed by saturated aqueous sodium bicarbonate until the pH reached 8–9. The resulting suspension was diluted with water (50 mL), stirred for 10 min at ambient temperature and left to stand at 5 °C for one hour. The product was collected by filtration, washed with water and dried in an oven at 100 °C to afford the title compound (1.15 g, 85%) as a tan solid (TLC: ethyl acetate/MeOH/AcOH, 98/2/0.2, R_f_ 0.69; ethyl acetate/hexane/AcOH, 80/20/0.2, R_f_ 0.57), mp 267 °C (dec.). The product was soluble in 0.5 N NaOH.

^1^H-NMR (400 MHz, DMSO-*d*_6_) δ 2.90 (t, 4H, *J* = 4.5 Hz, C*H*_2_NC*H*_2_), 3.72 (t, 4H, *J* = 4.5 Hz, C*H*_2_OC*H*_2_), 6.55 (d, 1H, *J* = 8.4 Hz, *H*^6^), 6.61 (d, 1H, *J* = 8.4 Hz, *H*^5^), 7.23 (s_br_, 2H, N*H*_2_), 8.89 (s, 1H, O*H*). ^13^C-NMR (100 MHz, DMSO-*d*_6_) δ 51.8 (*C*N*C*), 67.1 (*C*O*C*), 111.3 (*C*^5^), 112.1 (*C*^6^), 126.2 (*C*^7a^), 138.8 (*C*^3a^), 142.3 (*C*^7^), 144.5 (*C*^4^), 165.1 (*C*^2^). Anal. calcd for C_11_H_13_N_3_O_2_S: C, 52.57; H, 5.21; N, 16.72; found: C, 52.55; H, 5.17; N, 16.74. HRMS *m*/*z*: [M + H]^+^ calcd: 252.0801; found: 252.0796.

#### 3.2.2. Preparation of (*E*)-*N*′-(4-Hydroxy-7-morpholinobenzo[*d*]thiazol-2-yl)-*N*,*N*-dimethylformamidine (**3**)


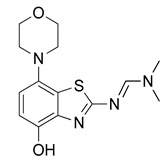


*N*,*N*-Dimethylformamide dimethyl acetal (160 µL, 1.2 mmol) was added to a solution of **2** (251 mg, 1 mmol) in anhydrous DMF (7 mL) and the mixture was stirred at 60 °C for 30 min (TLC: ethyl acetate/hexane/AcOH, 80/20/0.2, R_fsm_ 0.56, R_fp_ 0.25–0.39 extended spot). The reaction mixture was cooled to ambient temperature, poured into ice/half-saturated brine (50:50, 100 mL) and stirred for 10 min. Crystallization was induced by scratching with a glass rod and the mixture was left to stand at 5 °C overnight. The resulting precipitate was collected by filtration and dried at 100 °C to afford the title compound (280 mg, 91%) as off-white crystals, mp 244 °C (dec.).

^1^H-NMR (400 MHz, DMSO-*d*_6_) δ 2.94 (t, 4H, *J* = 4.5 Hz, C*H*_2_NC*H*_2_), 3.02 (s, 3H, N-C*H*_3_), 3.16 (s, 3H, N-C*H*_3_), 3.73 (t, 4H, *J* = 4.5 Hz, C*H*_2_OC*H*_2_), 6.56 (d, 1H, *J* = 8.4 Hz, *H*^6^), 6.69 (d, 1H, *J* = 8.4 Hz, *H*^5^), 8.38 (s, 1H, N-C*H*), 9.27 (s, 1H, O*H*). ^13^C-NMR (100 MHz, DMSO-*d*_6_) δ 35.1 (N-*C*H_3_), 40.8 (N-*C*H_3_), 51.8 (*C*N*C*), 67.1 (*C*O*C*), 111.7 (*C*^5^), 112.9 (*C*^6^), 127.6 (*C*^7a^), 138.8 (*C*^3a^), 141.8 (*C*^7^), 146.0 (*C*^4^), 158.1 (N-*C*H), 170.7 (*C*^2^). Anal. calcd for C_14_H_18_N_4_O_2_S: C, 54.88; H, 5.92; N, 18.29; found: C, 54.86; H, 5.89; N, 18.31. HRMS *m*/*z*: [M + H]^+^ calcd: 307.1223; found: 307.1220.

#### 3.2.3. Preparation of (*E*)-*N*′-(4-Benzyloxy-7-morpholinobenzo[*d*]thiazol-2-yl)-*N*,*N*-dimethylformamidine (**4**)


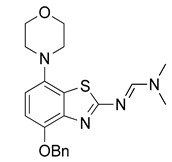


Cs_2_CO_3_ (489 mg, 1.5 mmol) was added, followed by benzyl bromide (155 µL, 1.3 mmol), under argon to a stirred solution of **3** (306 mg, 1 mmol) in anhydrous DMF (5 mL). The dark solution was stirred for 2 h at ambient temperature (TLC: ethyl acetate/hexane/AcOH, 80/20/0.2, R_fsm_ 0.25–0.39, R_fp_ 0.54), poured into ice/half-saturated brine (50:50, 100 mL), stirred for 10 min and cooled to 5 °C for 1 h. The resulting gelatinous precipitate was collected by filtration (40 mm diam. filter funnel!) and dried at 100 °C to afford the title compound (290 mg, 73%) as a dark solid. Recrystallization from 2-propanol (25 mL/g, some dark insoluble material remained) furnished the product as chocolate-colored brown crystals, mp 272 °C (dec.).

^1^H-NMR (400 MHz, DMSO-*d*_6_) δ 2.98 (t, 4H, *J* = 4.5 Hz, C*H*_2_NC*H*_2_), 3.03 (s, 3H, N-C*H*_3_), 3.17 (s, 3H, N-C*H*_3_), 3.74 (t, 4H, *J* = 4.5 Hz, C*H*_2_OC*H*_2_), 5.22 (s, 2H, C_6_H_5_-C*H*_2_), 6.74 (d, 1H, *J* = 8.4 Hz, *H*^6^), 6.91 (d, 1H, *J* = 8.4 Hz, *H*^5^), 7.30–7.36 (m, 1H, C_6_H_5_-*H*^4^), 7.43–7.37 (m, 2H, C_6_H_5_-*H*^3^), 7.46–7.51 (m, 2H, C_6_H_5_-*H*^2^), 8.40 (s, 1H, N-C*H*). ^13^C-NMR (100 MHz, DMSO-*d*_6_) δ 35.1 (N-*C*H_3_), 40.8 (N-*C*H_3_), 51.6 (*C*N*C*), 67.0 (*C*O*C*), 70.9 (C_6_H_5_-*C*H_2_), 110.8 (*C*^5^), 112.2 (*C*^6^), 127.9 (*C*^7a^), 128.2 (C_6_H_5_-*C*^2,4,6^), 128.8 (C_6_H_5_-*C*^3,5^), 138.0 (C_6_H_5_-*C*^1^), 140.6 (*C*^3a^), 143.1 (*C*^7^), 147.0 (*C*^4^), 157.9 (N-*C*H), 171.8 (*C*^2^). Anal. calcd for C_21_H_24_N_4_O_2_S: C, 63.61; H, 6.10; N, 14.13; found: C, 63.60; H, 6.06; N, 14.11. HRMS *m*/*z*: [M + H]^+^ calcd: 397.1693; found: 397.1689.

#### 3.2.4. Preparation of 4-Benzyloxy-7-morpholinobenzo[*d*]thiazol-2-amine (**5**)


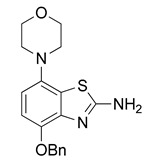


2 N aqueous KOH (2 mL, 4 mmol) was added to a well-stirred suspension of **4** (396 mg, 1 mmol) in MeOH (8 mL) and the suspension was stirred at 80 °C for 1 h (TLC: ethyl acetate/hexane/AcOH, 80/20/0.2, R_fsm_ 0.54, R_fp_ 0.85), after which water (8 mL) was added and the mixture cooled to ambient temperature. The precipitate was collected by filtration and dried at 100 °C to afford the title compound (277 mg, 81%) as fawn solid, mp 208 °C (dec.).

^1^H-NMR (400 MHz, DMSO-*d*_6_) δ 3.04 (t, 4H, *J* = 4.5 Hz, C*H*_2_NC*H*_2_), 3.88 (t, 4H, *J* = 4.5 Hz, C*H*_2_OC*H*_2_), 5.15 (s, 2H, C_6_H_5_-C*H*_2_), 6.66 (d, 1H, *J* = 8.4 Hz, *H*^6^), 6.79 (d, 1H, *J* = 8.4 Hz, *H*^5^), 7.29–7.35 (m, 1H, C_6_H_5_-*H*^4^), 7.36–7.42 (m, 2H, C_6_H_5_-*H*^3^), 7.43–7.49 (m, 2H, C_6_H_5_-*H*^2^). ^13^C-NMR (100 MHz, DMSO-*d*_6_) δ 51.7 (*C*N*C*), 67.0 (*C*O*C*), 70.9 (C_6_H_5_-*C*H_2_), 109.0 (*C*^5^), 111.0 (*C*^6^), 126.2 (*C*^7a^), 128.1 (C_6_H_5_-*C*^2,4,6^), 128.8 (C_6_H_5_-*C*^3,5^), 138.1 (C_6_H_5_-*C*^1^), 140.6 (*C*^3a^), 142.7 (*C*^7^), 146.3 (*C*^4^), 166.1 (*C*^2^). Anal. calcd for C_18_H_19_N_3_O_2_S: C, 63.32; H, 5.61; N, 12.31; found: C, 63.34; H, 5.62; N, 12.28. HRMS *m*/*z*: [M + H]^+^ calcd: 342.1271; found: 342.1268.

#### 3.2.5. Preparation of Phenyl(4-benzyloxy)-7-morpholinobenzo[*d*]thiazol-2-yl)carbamate (**6**)


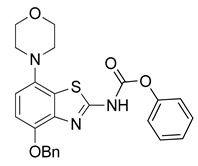


Pyridine (243 µL, 3 mmol) was added to a well-stirred and pre-cooled (0–5 °C, ice bath) suspension of **5** (341 mg, 1 mmol) in anhydrous THF (7.5 mL). After stirring for 5 min, phenyl chloroformate (144 µL, 1.15 mmol) was slowly added and the mixture was stirred for another 5 min. The ice bath was removed, the reaction mixture was stirred at ambient temperature for 0.5 h (TLC: sample in acetone **not** MeOH, ethyl acetate/hexane, 80/20, R_fs.m._ 0.70, R_fp_ 0.90) and then diluted with dichloromethane (50 mL) and water (15 mL). The organic layer was separated, washed with aqueous HCl (0.5 N, 50 mL) and brine (50 mL), dried over anhydrous Na_2_SO_4_ and concentrated under reduced pressure. Extraction of the residue with hot ethanol followed by cooling to ambient temperature, filtration and drying at 60 °C afforded the title compound (433 mg, 0.94 mmol, 94%) as porcelain-colored crystals, mp 229 °C (dec.).

^1^H-NMR (400 MHz, DMSO-*d*_6_) δ 3.00 (t, 4H, *J* = 4.5 Hz, C*H*_2_NC*H*_2_), 3.75 (t, 4H, *J* = 4.5 Hz, C*H*_2_OC*H*_2_), 5.24 (s, 2H, Bn-C*H*_2_), 6.89 (d, 1H, *J* = 8.5 Hz, *H*^6^), 7.05 (d, 1H, *J* = 8.5 Hz, *H*^5^), 7.26–7.54 (m, 10H, 2 × C_6_*H*_5_), 12.70 (s_br_, 1H, N*H*). ^13^C-NMR (100 MHz, DMSO-*d*_6_) δ 158.3 (*C*^2^), 51.9 (*C*N*C*), 67.0 (*C*O*C*), 70.7 (Bn-*C*H_2_), 110.3 (*C*^5^), 113.4 (*C*^6^), 127.4 (*C*^7a^), 126.6 (Bn-*C*^2,3,4 +^ Ph-*C*^2,3,4^), 136.8 (Bn-*C*^1^), 137.6 (Bn-*C*^1^), 140.6 (*C*^3a^), 145.7 (*C*^7^), 147.6 (*C*^4^), 153.1 (*C*=O). Anal. calcd for C_25_H_23_N_3_O_4_S: C, 65.06; H, 5.02; N, 9.10; found: C, 65.08; H, 5.00; N, 9.08. HRMS *m*/*z*: [M + H]^+^ calcd: 462.1482; found: 462.1480.

#### 3.2.6. Preparation of *N*-(4-(Benzyloxy)-7-morpholinobenzo[*d*]thiazol-2-yl)-4-hydroxy-4-methylpiperidine-1-carboxamide (**7**) [25]


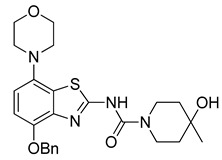


4-Hydroxy-4-methylpiperidine (126 mg, 1.08 mmol) was added to a stirred solution (prepared under argon) of **6** (461 mg, 1 mmol) in anhydrous DMSO (10 mL) and the clear brown mixture was stirred at 50 °C for 1 h (TLC: sample in acetone **not** MeOH, do **not** dry with a heat gun, ethyl acetate/hexane, 80/20, R_fCarbamate_ 0.90, R_fUrea_ 0.35, R_fPhenol_ 0.98). After cooling to ambient temperature, 50% brine (40 mL) was added and the resulting precipitate was extracted into ethyl acetate (2 × 50 mL). The organic phase was washed with 10% citric acid (50 mL), saturated aqueous Na_2_CO_3_ (50 mL), water and brine and dried over anhydrous Na_2_SO_4_. Concentration under reduced pressure produced a solid that was treated with boiling ethanol (ca. 7 mL) and cooled overnight. The resulting precipitate was collected by filtration and dried at 60 °C to afford the title compound (400 mg, 83%) as beige crystals, mp 210 °C.

^1^H-NMR (400 MHz, DMSO-*d*_6_) δ 1.14 (s, 3H, C*H*_3_), 1.35–1.53 (m, 4H, Pip-C^3^*H*_2_ + Pip-C^5^*H*_2_), 3.00 (t, 4H, *J* = 4.5 Hz, C*H*_2_NC*H*_2_), 3.19–3.32 (m, 2H, 0.5 Pip-C^2^*H*_2_ + 0.5 Pip-C^6^*H*_2_), 3.76 (t, 4H, *J* = 4.5 Hz, C*H*_2_OC*H*_2_), 3.79–3.90 (m, 2H, 0.5 Pip-C^2^*H*_2_ + 0.5 Pipc^6^*H*_2_), 4.42 (s_br_, 1H, O*H*), 5.17 (s, 2H, Bn-C*H*_2_), 6.78 (d, 1H, *J* = 8.5 Hz, *H*^6^), 6.96 (d, 1H, *J* = 8.5 Hz, *H*^5^), 7.30–7.55 (m, 5H, C_6_*H*_5_), 12.21 (s_br_, 1H, N*H*). ^13^C-NMR (100 MHz, DMSO-*d*_6_) δ 30.3 (*C*H_3_), 38.7 (Pip-*C*^3,5^), 40.8 (Pip-*C*^2,6^), 51.9 (*C*N*C*), 66.5 (Pip-*C*^4^), 67.1 (*C*O*C*), 70.7 (Bn-*C*H_2_), 109.7 (*C*^5^), 112.4 (*C*^6^), 127.4 (*C*^7a^), 128.9, 128.7, 128.4, (Bn-*C*^2,3,4^), 137.6 (Bn-*C*^1^), 140.4 (*C*^3a^), 147.3 (*C*^7^), 150.7 (*C*^4^), 153.7 (*C*=O), 160.4 (*C*^2^). Anal. calcd for C_25_H_30_N_4_O_4_S: C, 62.22; H, 6.27; N, 11.61; found: C, 62.20; H, 6.23; N, 11.56. HRMS *m*/*z*: [M + H]^+^ calcd: 483.2061; found: 483.2055.

#### 3.2.7. Preparation of 4-Hydroxy-*N*-(4-hydroxy-7-morpholinobenzo[*d*]thiazol-2-yl)-4-methylpiperidine-1-carboxamide (**8**) [34]


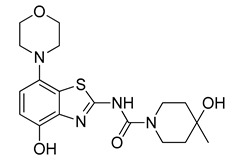


Ammonium formate (380 mg, 6 mmol) followed by 10% Pd/C (300 mg, 0.3 mmol) were added to a stirred solution of **7** (482 mg, 1 mmol) in anhydrous DMF (5 mL). The flask was sealed with a bubble counter and immersed in a pre-heated (55 °C) oil bath. The reaction mixture was stirred at 55 °C for ten min (TLC: ethyl acetate/hexane, 80/20, R_fs.m._ 0.42, R_fp_ 0.08–0.2, extended spot), cooled to ambient temperature and filtered through a layer of Quartz/Celite (prewetted with DMF). The filter cake was washed with DMF, the combined filtrate and washings were poured into ice/half-saturated brine (50:50, 50 mL) and the resulting mixture was stirred for 10 min. Crystallization was induced by scratching with a glass rod and the mixture was cooled to 5 °C overnight. The solid was collected by filtration, washed with MeOH and dried at 50 °C to afford the title compound (290 mg, 74%) as a light grey solid. For further purification, the solid was dissolved in a minimum amount of hot DMF and the same volume of water was added. The nearly colorless precipitate formed upon cooling was collected by filtration and treated with boiling MeOH. After cooling to ambient temperature, the solid was collected by filtration, washed with MeOH and dried at 50 °C, mp 265 °C (dec.).

^1^H-NMR (400 MHz, DMSO-*d*_6_) δ 1.15 (s, 3H, C*H*_3_), 1.57–1.33 (m, 4H, Pip-C^3^*H*_2_ + Pip-C^5^*H*_2_), 2.96 (t, 4H, *J* = 4.5 Hz, C*H*_2_NC*H*_2_), 3.19–3.32 (m_obsc_, 2H, 0.5 Pip-C^2^*H*_2_ + 0.5 Pip-C^6^*H*_2_), 3.76 (t, 4H, *J* = 4.5 Hz, C*H*_2_OC*H*_2_), 3.80–3.96 (m, 2H, 0.5 Pip-C^2^*H*_2_ + 0.5 Pipc^6^*H*_2_), 4.39 (s_br_, 1H, O*H*), 6.66–6.76 (m, 2H, *H*^5^, *H*^6^), 9.37 (s_br_, 1H, O*H*), 11.16 (s_br_, 1H, N*H*). ^13^C-NMR (100 MHz, DMSO-*d*_6_) δ 30.2 (*C*H_3_). 38.6 (Pip-*C*^3,5^), 40.8 (Pip-*C*^2,6^), 51.8 (*C*N*C*), 66.5 (Pip-*C*^4^), 67.0 (*C*O*C*), 109.5 (*C*^5^), 112.2 (*C*^6^), 127.1 (*C*^7a^), 140.3 (*C*^3a^), 140.5 (*C*^7^), 147.1 (*C*^4^), 153.6 (*C*=O), 160.7 (*C*^2^). Anal. calcd for C_18_H_24_N_4_O_4_S: C, 55.08; H, 6.16; N, 14.28; found: C, 54.99; H, 6.11; N, 14.31. HRMS *m*/*z*: [M + H]^+^ calcd: 393.1591; found: 393.1588.

#### 3.2.8. Preparation of Tetrabutylammonium 2-(4-Hydroxy-4-methylpiperidine-1-carboxamido)-7-morpholinobenzo[*d*]thiazol-4-olate (**8a**) (According to a Modification of the Procedure in [27])


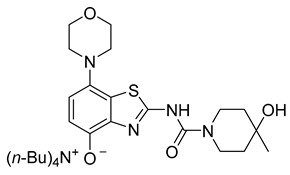


Tetrabutylammonium hydroxide (1 m solution in MeOH, 100 µL, 100 µmol, 1 equiv) was added dropwise to a vortexed suspension of **8** (47 mg, 120 µmol, 1.2 equiv) in MeOH (2.4 mL, 20 µL/µmol **8**, *c* = 0.05 m). The mixture was vortexed for 1 min and then centrifuged at 20,000 rpm for 1 min to remove unreacted **8**. The clear yellow supernatant was recovered with a pipette and MeOH evaporated under reduced pressure. The product was dried using careful heating (<40 °C) under reduced pressure to afford the title compound (58 mg, 92% yield based on TBAOH) as a greenish semi-solid.

^1^H-NMR (400 MHz, CDCl_3_) δ = 0.90 (t, *J* = 7.3 Hz, 12H, TBAC^4^*H*_3_), 1.21–1.30 (m, 11H, TBAC^3^*H*_2_ + C*H*_3_), 1.34–1.42 (m, 8H, TBAC^2^*H*_2_), 1.57 (t, *J* = 5.5 Hz, 4H, Pip-C^3^*H*_2_ + Pip-C^5^*H*_2_), 3.08 (s_br_, 4H, C*H*_2_NC*H*_2_), 2.87–2.91 (m, 8H, TBAC^1^*H*_2_), 3.46–3.50 (m, 3H, 0.5 Pip-C^2^*H*_2_ + 0.5 Pip-C^6^*H*_2_ + O*H*), 3.82 (t, 4H, *J* = 4.3 Hz, C*H*_2_OC*H*_2_), 4.05 (s_br_, 2H, 0.5 Pip-C^2^*H*_2_ + 0.5 Pipc^6^*H*_2_), 6.38 (d, *J* = 8.2 Hz, 1H, *H*^6^), 6.88 (d, *J* = 8.2 Hz, 1H, *H*^5^). ^13^C-NMR (101 MHz, CDCl_3_) δ = 13.7 (TBA*C*^4^), 19.5 (TBA*C*^3^), 23.8 (TBA*C*^2^),29.6 (*C*H_3_), 39.1 (Pip-*C*^3,5^), 40.1 (Pip-*C*^2,6^), 51.5 (*C*N*C*), 58.3 (TBA*C*^1^), 67.6 (*C*O*C*), 68.7 (Pip-*C*^4^), 109.6 (*C*^6^), 111.9 (*C*^5^), 124.7 (*C*^7a^), 137.8 (*C*^3a^), 139.5 (*C*^7^), 142.2 (*C*^4^), 162.3 (*C*=O), 170.8 (*C*^2^). C_34_H_59_N_5_O_4_S, 633.92 g/mol. MS not possible due to decomposition.

#### 3.2.9. Preparation of (*N*-(4-(Benzyloxy)-7-morpholinobenzo[*d*]thiazol-2-yl)-4-hydroxy-4-methylpiperidine-1-carboxamido)methyl Pivalate (**9**)


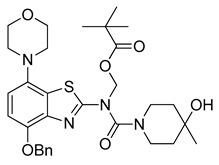


Anhydrous potassium carbonate (207 mg, 1.5 mmol) was added under argon to a stirred solution of **7** (482 mg, 1 mmol) in anhydrous DMF (20 mL). The mixture was stirred at 60 °C for 30 min and a solution of chloromethyl pivalate (252 µL, 1.3 mmol) in anhydrous DMF (500 µL) was slowly added. After stirring for another 1 h at 60 °C (TLC: ethyl acetate/hexane, 70/30, R_fUrea_ 0.37, R_fPom-urea_ 0.44), the reaction mixture was cooled to ambient temperature and poured into a combination of ice/water (150 mL). The mixture was stirred for 0.5 h and cooled overnight. The resulting precipitate was collected via filtration and treated with boiling ethyl acetate (± 25 mL). After cooling to ambient temperature, the solid was recovered by filtration and dried at 60 °C to afford the title compound (555 mg, 91%) as colorless crystals, mp >300 °C (ethyl acetate).

^1^H-NMR (400 MHz, DMSO-*d*_6_) δ 1.03 (s, 9H, Pom-C*H*_3_), 1.13 (s, 3H, C*H*_3_), 1.27–1.53 (m, 4H, Pip-C^3^*H*_2_ + Pip-C^5^*H*_2_), 2.95 (t, 4H, *J* = 4.5 Hz, C*H*_2_NC*H*_2_), 3.12–3.28 (m, 2H, 0.5 Pip-C^2^*H*_2_ + 0.5 Pip-C^6^*H*_2_), 3.76 (t, 4H, *J* = 4.5 Hz, C*H*_2_OC*H*_2_), 3.84 + 4.04 (2s_br_, 2H, 0.5 Pip-C^2^*H*_2_ + 0.5 PipC^6^*H*_2_), 4.36 (s_br_, 1H, O*H*), 5.22 (s, 2H, Bn-C*H*_2_), 6.43 (s_br_, 2H, Pom-C*H*_2_), 6.94 (d, 1H, *J* = 8.5 Hz, *H*^6^), 7.17 (d, 1H, *J* = 8.5 Hz, *H*^5^), 7.30–7.50 (m, 5H, C_6_*H*_5_). ^13^C-NMR (100 MHz, DMSO-*d*_6_) δ 27.0 (Pom C*H*_3_), 30.7 (*C*H_3_), 38.7 (Pip-*C*^3,5^), 40.9 (Pip-*C*^2,6^), 52.0 (*C*N*C*), 66.7 (Pip-*C*^4^), 67.0 (*C*O*C*), 70.4 (Bn-*C*H_2_), 71.2 (Pom *C*H_2_), 112.5 (*C*^5^), 114.2 (*C*^6^), 122.0 (Bn-*C*^1^), 125.6 (*C*^7a^), 128.9, 128.5, 127.9, (Bn-*C*^2,3,4^), 136.9 (*C*^3a^), 141.0 (*C*^7^), 142.3 (*C*^4^), 160.1 (*C*=O), 166.2 (*C*^2^), 176.9 (Pom *C*=O). Anal. calcd for C_31_H_40_N_4_O_6_S: C, 62.39; H, 6.76; N, 9.39; found: C, 62.44; H, 6.69; N, 9.40. HRMS *m*/*z*: [M + H]^+^ calcd: 597.2741; found: 597.2749.

#### 3.2.10. Preparation of (*N*-(4-(Hydroxy)-7-morpholinobenzo[*d*]thiazol-2-yl)-4-hydroxy-4-methylpiperidine-1-carboxamido)methyl Pivalate (**10**)


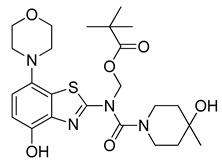


Ammonium formate (315 mg, 5 mmol) was added to a stirred suspension of **9** (291 mg, 0.5 mmol) in MeOH and AcOH (50:50, 5 mL), followed by 10% Pd/C (150 mg). The flask was sealed with a bubble counter and immersed in a pre-heated (55 °C) oil bath. The mixture was stirred at 55 °C for 10 min (evolution of hydrogen gas started after 2 min; TLC: ethyl acetate/hexane, 80/20, R_fs.m._ 0.51, R_fp_ 0.41), cooled to ambient temperature and filtered through a layer of Quartz/Celite (prewetted with MeOH). The filter cake was washed with MeOH and the combined filtrate and washings were concentrated under reduced pressure at 40–45 °C to remove most of the MeOH. The remaining solution was poured into a combination of ice/water (50:50, 200 mL) and the resulting mixture was stirred for 10 min and then extracted with dichloromethane (2 × 75 mL). The combined extracts were washed with saturated aqueous sodium bicarbonate (50 mL) and brine (100 mL) and dried over anhydrous Na_2_SO_4_. Subsequent evaporation of the solvent afforded the analytically pure title compound (187 mg, 74%) as a colorless powder, mp 182 °C.

^1^H-NMR (400 MHz, DMSO-*d*_6_) δ 1.11 (s, 9H, C(C*H*_3_)_3_), 1.14 (s, 3H, C*H*_3_), 1.31–1.56 (m, 4H, Pip-C^3^*H*_2_ + Pip-C^5^*H*_2_), 2.90 (t, 4H, *J* = 4.5 Hz, C*H*_2_NC*H*_2_), 3.17–3.45 (m, 2H, 0.5 Pip-C^2^*H*_2_ + 0.5 Pip-C^6^*H*_2_), 3.74 (t, 4H, *J* = 4.5 Hz, C*H*_2_OC*H*_2_), 3.80–4.14 (m, 2H, 0.5 Pip-C^2^*H*_2_ + 0.5 Pipc^6^*H*_2_), 4.35 (s_br_, 1H, O*H*), 6.46 (s, 2H, C*H*_2_-Pom), 6.82 (s_br_, 2H, *H*^5^, *H*^6^), 10.18 (s_br_, 1H, O*H*). ^13^C-NMR (100 MHz, DMSO-*d*_6_) δ 27.2 (C(*C*H_3_)_3_), 30.1 (*C*H_3_). 38.6 (Pip-*C*^3,5^), 40.9 (Pip-*C*^2,6^), 52.2 (*C*N*C*), 66.7 (Pip-*C*^4^), 67.0 (*C*O*C*), 69.9 (*C*(CH_3_)_3_), 114.4 (*C*^5^), 114.7 (*C*^6^), 122.2 (*C*^7a^), 124.2 (*C*^3a^), 139.4 (*C*^7^), 141.1 (*C*^4^), 160.2 (*C*=O), 166.2 (*C*^2^), 177.2 (Pom C=O). Anal. calcd for C_24_H_34_N_4_O_6_S: C, 56.90; H, 6.76; N, 11.06; found: C, 56.86; H, 6.72; N, 10.94. HRMS *m*/*z*: [M + H]^+^ calcd: 507.2272; found: 507.2270.

#### 3.2.11. Preparation of 2-(4-Hydroxy-4-methylpiperidine-1-carboxamido)-7-morpholinobenzo[*d*]thiazol-4-yl Pivalate (**10a**)


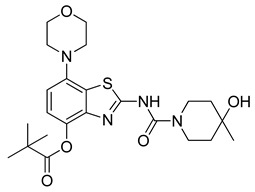


Ammonium formate (315 mg, 5 mmol) was added to a stirred solution of **9** (291 mg, 0.5 mmol) in DMF (5 mL), followed by 10% Pd/C (150 mg). The flask was sealed with a bubble counter and immersed in a pre-heated (55 °C) oil bath. The mixture was stirred at 55 °C for 10 min (TLC: ethyl acetate/hexane, 80/20, R_fs.m._ 0.51, R_fp_ 0.41), cooled to ambient temperature and filtered through a layer of Quartz/Celite (prewetted with DMF). The filter cake was washed with DMF, and the combined filtrate and washings were poured into a combination of ice/water (50:50, 200 mL). The resulting mixture was stirred for 10 min and then extracted with ethyl acetate (2 × 75 mL). The combined extracts were washed with saturated aqueous sodium bicarbonate (50 mL) and brine (100 mL) and dried over anhydrous Na_2_SO_4_. Subsequent evaporation of the solvent furnished a solid, which was recrystallized from 80% aqueous MeOH to afford the analytically pure title compound (187 mg, 74%) as a light grey solid, mp 246 °C.

^1^H-NMR (400 MHz, DMSO-*d*_6_) δ 1.16 (s, 3H, C*H*_3_), 1.37 (s, 9H, C(C*H*_3_)_3_), 1.40–1.58 (m, 4H, Pip-C^3^*H*_2_ + Pip-C^5^*H*_2_), 3.08 (t, 4H, *J* = 4.5 Hz, C*H*_2_NC*H*_2_), 3.21–3.36 (m, 2H, 0.5 Pip-C^2^*H*_2_ + 0.5 Pipc^6^*H*_2_), 3.79 (t, 4H, *J* = 4.5 Hz, C*H*_2_OC*H*_2_), 3.76–3.91 (m, 2H, 0.5 Pip-C^2^*H*_2_ + 0.5 Pip-C^6^*H*_2_), 4.40 (s_br_, 1H, O*H*), 6.83 (d, 2H, *H*^6^), 6.99 (d, 2H, *H*^5^), 10.93 (s, 1H, N*H*). ^13^C-NMR (100 MHz, DMSO-*d*_6_) δ 27.4 (C(*C*H_3_)_3_), 30.1 (*C*H_3_). 38.5 (Pip-*C*^3,5^), 39.0 (*C*(CH_3_)_3_), 40.9 (Pip-*C*^2,6^), 51.5 (*C*N*C*), 66.3 (Pip-*C*^4^), 66.8 (*C*O*C*), 111.8 (*C*^6^), 119.6 (*C*^5^), 130.5 (*C*^7a^), 141.9 (*C*^3a^), 144.3 (*C*^7^), 146.3 (*C*^4^), 150.9 (*C*=O), 160.3 (*C*^2^), 177.3 (Piv C=O). Anal. calcd for C_23_H_32_N_4_O_5_S: C, 57.96; H, 6.77; N, 11.76; found: C, 57.93; H, 6.79; N, 11.77. HRMS *m*/*z*: [M + H]^+^ calcd: 477.2166; found: 477.2158.

#### 3.2.12. Preparation of *tert*-Butyl (4-(benzyloxy)-7-morpholinobenzo[*d*]thiazol-2-yl)(4-hydroxy-4-methylpiperidine-1-carbonyl)carbamate (**11**)


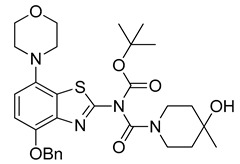


Triethylamine (210 µL, 3 equiv) and di-*tert*-butyl dicarbonate (218 mg, 1 mmol, 2 equiv) were added to a well-stirred suspension of **7** (241 mg, 0.5 mmol, 1 equiv) in anhydrous THF (6 mL). 4-(Dimethylamino)pyridine (61 mg, 0.5 mmol, 0.5 equiv) was added and the reaction mixture was stirred for 10 min (TLC: ethyl acetate/hexane, 80/20, R_fs.m._ 0.35, R_fp_ 0.76), during which the color of the solution changed to orange and then yellow. The reaction was quenched by addition of saturated aqueous sodium bicarbonate (10 mL), and the resulting mixture was extracted with dichloromethane (2 × 40 mL). The combined extracts were washed with 1% aqueous HCl (50 mL), water (50 mL) and brine (50 mL) and dried over anhydrous Na_2_SO_4_. Subsequent evaporation of the solvent under reduced pressure afforded the title compound (260 mg, 89%) as off-white crystals, mp 91 °C.

^1^H-NMR (400 MHz, DMSO-*d*_6_) δ 1.06 (s_br_, 3H, C*H*_3_), 1.43–1.63 (m, 4H, Pip-C^3^*H*_2_ + Pip-C^5^*H*_2_), 1.52 (s, 9H, Boc-C*H*_3_), 3.03 (t, 4H, *J* = 4.5 Hz, C*H*_2_NC*H*_2_), 3.25–3.44 (m, 3H, 0.5 Pip-C^2^*H*_2_ + 0.5 Pip-C^6^*H*_2_ + 0.5 Pip-C^2/6^*H*_2_), 3.78 (t, 4H, *J* = 4.5 Hz, C*H*_2_OC*H*_2_), 3.84 + 4.04 (s_br_, 1H, 0.5 Pip- C^2/6^*H*_2_), 4.51 (s_br_, 1H, O*H*), 5.26 (s, 2H, Bn-C*H*_2_), 6.94 (d, 1H, *J* = 8.5 Hz, *H*^6^), 7.03 (d, 1H, *J* = 8.5 Hz, *H*^5^), 7.28–7.50 (m, 5H, C_6_*H*_5_). ^13^C-NMR (100 MHz, DMSO-*d*_6_) δ 28.0 (Boc C*H*_3_), 30.2 (*C*H_3_), 51.9 (*C*N*C*), 66.0 (Pip-*C*^4^), 67.0 (*C*O*C*), 71.3 (Bn-*C*H_2_), 85.1 (Boc *C*), 114.1 (*C*^5/6^), 122.0 (Bn-*C*^1^), 125.6 (*C*^7a^), 127.8, 128.2, 128.8, (Bn-*C*^2,3,4^), 137.8 (*C*^4a^), 140.8 (*C*^7^), 147.6 (*C*^4^), 149.0 (*C*^2^), 150.3 (Boc *C*=O), 156.3 (Pip *C*=O)(signals for the piperidine methylene carbons are missing). Anal. calcd for C_30_H_38_N_4_O_6_S: C, 61.84; H, 6.57; N, 9.61; found: C, 61.79; H, 6.61; N, 9.57. HRMS *m*/*z*: [M + H]^+^ calcd: 583.2585; found: 583.2583.

#### 3.2.13. Preparation of *tert-*Butyl (4-hydroxy-4-methylpiperidine-1-carbonyl)(4-hydroxy-7-morpholinobenzo[*d*]thiazol-2-yl)carbamate (**12**)


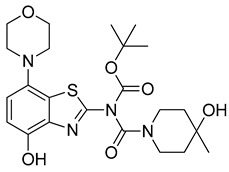


Ammonium formate (126 mg, 2 mmol) was added to a stirred solution of **11** (116 mg, 0.2 mmol) in anhydrous DMF (4 mL), followed by 10% Pd/C (50 mg). The flask was sealed with a bubble counter and immersed in a pre-heated (55 °C) oil bath. The mixture was stirred at 55 °C for 20 min (rapid evolution of hydrogen gas started after 2 min; TLC: ethyl acetate/hexane, 80/20, R_fs.m._ 0.76, R_fp_ 0.51), cooled to ambient temperature and filtered through a layer of Quartz/Celite (prewetted with DMF). The filter cake was washed with DMF, and the combined filtrate and washings were poured into ice/half-saturated brine (50:50, 50 mL). The resulting mixture was stirred for 10 min and then extracted with ethyl acetate (2 × 30 mL). The combined extracts were washed with brine and dried over anhydrous Na_2_SO_4_. Evaporation of the solvent afforded a semi-solid residue, which was crystallized by co-evaporation with methyl *tert*-butyl ether to obtain the crude product as slightly yellow crystals. Further purification by flash chromatography (ethyl acetate/hexane, 50/50) afforded the title compound (97 mg, 98%) as colorless crystals, mp 259 °C (dec).

^1^H-NMR (400 MHz, DMSO-*d*_6_) δ 1.16 (s_br_, 3H, C*H*_3_), 1.42–1.69 (m, 4H, Pip-C^3^*H*_2_ + Pip-C^5^*H*_2_), 1.53 (s, 9H, Boc-C*H*_3_), 2.97 (t, 4H, *J* = 4.5 Hz, C*H*_2_NC*H*_2_), 3.25–3.44 (m_obsc_., 3H, 0.5 Pip-C^2^*H*_2_ + 0.5 Pip-C^6^*H*_2_ + 0.5 Pip-C^2/6^*H*_2_), 3.77 (t, 4H, *J* = 4.5 Hz, C*H*_2_OC*H*_2_), 4.01 (s_br_, 1H, 0.5 Pip- C^2/6^*H*_2_), 4.55 (s_br_, 1H, O*H*), 6.80 (d, 1H, *J* = 8.5 Hz, *H*^6^), 6.85 (d, 1H, *J* = 8.5 Hz, *H*^5^), 9.55 (s, 1H, O*H*). ^13^C-NMR (100 MHz, DMSO-*d*_6_) δ 28.1 (Boc C*H*_3_), 30.4 (*C*H_3_), 37.9 (Pip-*C*H_2_), 38.3 (Pip-*C*H_2_), 43.4(Pip-*C*H_2_), 52.1 (*C*N*C*), 66.2 (Pip-*C*^4^), 67.1 (*C*O*C*), 84.9 (Boc *C*), 112.3 (*C*^6^), 114.6 (*C*^5^), 128.2 (*C*^7a^), 138.8 (*C*^4a^), 138.9 (*C*^7^), 146.8 (*C*^4^), 149.1 (*C*^2^), 150.4 (Boc *C*=O), 156.4 (Pip *C*=O).

### 3.3. Radiochemistry

#### 3.3.1. General Radiochemistry

[^11^C]CO_2_ (20–25 GBq) was produced via the ^14^N(p,α)^11^C nuclear reaction by irradiating (30–35 µA for 25–40 min) 99.5%_mol_ N_2_ doped with 0.5%_mol_ O_2_ in a GE PETtraceTM 800 cyclotron (GE Healthcare GmbH, Munich, Germany). Production of [^11^C]MeI was performed on a custom-built synthesizer as reported previously [35]. Briefly, [^11^C]CO_2_ was reduced to [^11^C]MeOH using 1 m LiAlH_4_ in THF (ABX, Radeberg, Germany) and the THF was removed at 100 °C in a stream of helium. After the addition of 4.3 m H_3_PO_4_, the [^11^C]MeOH was distilled off at 150 °C and passed through a pre-heated (170 °C) column with alumina-supported triphenylphosphine diiodide. The resulting [^11^C]MeI was passed through a second column with phosphorus pentoxide and collected in a reaction vial containing the labelling precursor (**8**,**10** or **12**) and the corresponding solvent pre-cooled to −10 °C (DMF, DMA) or 20 °C (DMSO), respectively. [^11^C]Methyl triflate ([^11^C]MeOTf) was prepared on a Synthra MeIplus system (Synthra GmbH, Hamburg, Germany) using the process and chemicals specified by the manufacturer. Briefly, [^11^C]CO_2_ produced as described above was converted to [^11^C]methane over nickel at 400 °C, followed by conversion to [^11^C]MeI by I_2_ in the gas phase at 700 °C in a continuous flow process where [^11^C]MeI was trapped on a Porapak Q column. Conversion to [^11^C]MeOTf was achieved via reaction with silver triflate at 190 °C. [^11^C]MeOTf was collected in a reaction vial containing precursor **8a** and solvent (acetone or acetonitrile) pre-cooled to −10 °C. The syntheses were performed in a helium atmosphere.

#### 3.3.2. Manual Screening Reactions for the Preparation of [^11^C]tozadenant

Manual methylation reactions with [^11^C]MeI were performed under air in 2 mL Eppendorf safe lock tubes. The vials were heated in thermoshakers with gentle agitation. The tubes were charged (in this order) with precursor **12** (1 mg, 2 µmol), the corresponding solvent (300 µL), 1 m aqueous NaOH (4 µmol) and a stock solution of [^11^C]MeI (5–10 MBq) in DMSO. The reaction mixture was heated at 60 °C for 5 min and cooled to 0 °C before the addition of HCl (37%, 150 µL). The mixture was then heated for another 5 min at 100 °C, cooled to 0 °C and diluted with 1 m ammonium formate (1.5 mL). The resulting crude product solution was spiked with the reference compound to identify the desired product and analyzed by analytical radio HPLC.

#### 3.3.3. Analytical HPLC

Samples prepared in manual radiolabeling experiments were analyzed on two different analytical HPLC systems (Knauer, Berlin, Germany) using identical analytical columns (MultoKrom^®^ 100-5 C18 250 × 4.6 mm) and solvents (0–10 min: 32.5% MeCN_aq_ + 0.2% HCOOH, 10–15 min: 80% MeCN_aq_ + 0.2% HCOOH; flow rate: 1.5 mL/min). Each system was equipped with a UV-Detector coupled in series with a LB 500 NaI radioactivity detector (Berthold Technologies, Bad Wilbach, Germany). Since HPLC analysis with post-column injection demonstrated near-quantitative recovery of radioactivity from the column, RCCs were calculated based on the summed integral of all peaks [36]. To account for variable signal strengths and slight differences in the retention time between the systems/columns (ca. 6%), all chromatograms were normalized to the highest peak and the time scale was adjusted with an appropriate correction factor.

#### 3.3.4. Automated Production of [^11^C]tozadenant ([^11^C]**13**)

1 N aqueous NaOH (4 µL, 4 µmol) was added to a solution of precursor **12** (1 mg, 2 µmol) in anhydrous DMA (300 µL) and the resulting slightly yellow solution was transferred into a reactor. The reactor was cooled to -10 °C to trap [^11^C]MeI and then heated to 60 °C for 5 min. After the addition of concentrated aqueous HCl (12 N, 150 µL, 1.8 mmol), the reactor was heated to 100 °C for 5 min to deprotect the radiolabeled intermediate and then cooled to 30 °C. A total of 2 m aqueous ammonium acetate (700 µL, 1.4 mmol) was added and the crude product solution was purified by semi-preparative HPLC (column: Kromasil 120-5-C18 ace-EPS, 250 × 8 mm; eluent: MeOH/water, 60/40, *v*/*v*; flow rate: 4 mL/min; detection: UV at 254 nm, radioactivity). The effluent fraction containing the product was collected, diluted with water for injection (30 mL) and passed through a Strata X 33 C18 cartridge (30 mg, preconditioned with 2 mL ethanol followed by 2 mL sterile water). The cartridge was washed with sterile water (5 mL) and the product was eluted with ethanol (500 µL) and diluted with sterile water (4.5 mL). Filtration through a sterile filter into a sterile vial produced an injectable sterile solution of [^11^C]tozadenant ([^11^C]**13**) containing 10% ethanol in volume, which was used for biological experiments.

#### 3.3.5. Quality Control

QMP compliant quality control of the final tracer formulation was performed based on the following methods and systems. Radio-HPLC: Chromaster HPLC system (Hitachi, Düsseldorf, Germany) coupled in series with an fLumo luminescence detector (Berthold Technologies, Bad Wilbach, Germany), conditions: Kromasil^®^ 100-5-C18 250 × 4.6 mm, 60% MeOH, 1.0 mL/min. Gas chromatography (GC): Intuvo 9000 GC system (Agilent, Ratingen, Germany), conditions: Agilent DB-624 Ultra Inert 30 m × 0.25 mm × 1.4 µm GC column, helium flow: 0–4 min: 2 mL/min, 4–8.5 min: 5 mL/min; temperature: 0–3.5 min: 40 °C, 4.5–8.5 min: 100 °C. Appearance was confirmed visually while pH was determined with pH indicator strips.

### 3.4. In Vitro Autoradiography

Cryostat sections (20 µm) of rat and pig brains were cut at −18 °C, thaw-mounted onto microscope slides, air-dried, and stored at −80 °C until use. For autoradiography, the cryosections were dried at 37 °C, pre-incubated in 50 mm TRIS (pH 7.4, 1 mm EDTA) for 5 min at ambient temperature, and then incubated with 12 kBq/mL [^11^C]tozadenant (0.24 nM) in the same buffer supplemented with 100 µm guanosine-5′-[(β,γ)-imido]triphosphate (GppNHp) for 30 min at ambient temperature. Non-specific binding was determined in the presence of 1 µm ZM241385. After the incubation, the sections were dipped twice in ice-cold deionized water, rapidly dried in a stream of cold air and exposed on an imaging plate for 1 h. The imaging plate was analyzed with a phosphor imager (BAS 5000, Fujifilm, Düsseldorf, Germany) and tracer binding was quantified via 2D densitometry (AIDA Image Analysis software V5.1). Specific binding was calculated as the difference between total and non-specific binding (4 slices each).

## 4. Conclusions

In conclusion, we synthesized *N*-Boc protected *O*-desmethyltozadenant (**12**) as a suitable precursor for the preparation of [^11^C]tozadenant by methylation with [^11^C]MeI, which affords this radioligand in radiochemical yields, molar activities and (radio)chemical purities sufficient for preclinical in vivo studies. In addition, in vitro autoradiography in pig and rat brain slices confirmed A_2A_R-specific binding of [^11^C]tozadenant. In vivo biodistribution studies using PET imaging in healthy rats to determine the cerebral pharmacokinetics and in vivo brain distribution of [^11^C]tozadenant are currently ongoing.

## Figures and Tables

**Table 1:** Quality control parameters of the final [^11^C]tozadenant tracer formulation.

Parameter	Method	Unit	Specification	Result
Appearance	visually	-	clear, no particles	conform
Activity concentration at EOS	calculated	MBq/mL	≥30	172 ± 95
Radiochemical identity	HPLC	%	±5	0 ± 2
Radiochemical purity	HPLC	%	≥90	95.0 ± 2.8
Carrier identity	HPLC	%	±5	0 ± 2
Carrier concentration	HPLC	µg/mL	≤5.0	1.12 ± 0.48
Total concentration of unknown impurities ^a^	HPLC	µg/mL	≤5.0	0.16 ± 0.16
Methanol concentration	GC	mg/mL	≤1.0	0.06 ± 0.03
Ethanol concentration	GC	%_vol_	≤10	9.10 ± 0.49
Acetone concentration	GC	mg/mL	≤1.0	n.d.
Acetonitrile concentration	GC	mg/mL	≤0.41	n.d.
DMSO concentration	GC	mg/mL	≤1.0	n.d.
DMA concentration	GC	mg/mL	≤1.0	n.d.
pH	indicator	-	4.5–8.5	5.3 ± 0.6

^a^ Based on the molecular weight and extinction coefficient of tozadenant.

## Data Availability

The data presented in this study are available in article and supplementary material.

## Supplementary Materials

The following supporting information can be downloaded at: https://www.mdpi.com/article/10.3390/molecules29051089/s1, ^1^H-, ^13^C- and ^19^F-NMR spectra of prepared compounds; HPLC and GC chromatograms of [^11^C]tozadenant (Figures S1–S3).
