# Supplementary material for: Radiosynthesis and In Vitro Evaluation of [11C]tozadenant as Adenosine A2A Receptor Radioligand
Source: Molecules. 2024 Feb 29;29(5):1089. doi: 10.3390/molecules29051089 (PMC10935082; doi:10.3390/molecules29051089)
Supplement: Supplementary file 1 [file molecules-29-01089-s001.zip › molecules-2885269-supplementary.pdf]

Electronic supporting material (ESI) for

Radiosynthesis and In Vitro Evaluation of [^{11}C]tozadenant as Adenosine A_{2A} Receptor Radioligand

Swen Humpert [1], Daniela Schneider [1], Markus Lang [1], Anette Schulze [1], Felix Neumaier [1,2], Marcus Holschbach [1], Dirk Bier [1], Bernd Neumaier [1,2,*]

[1] Forschungszentrum Jülich GmbH, Institute of Neuroscience and Medicine, Nuclear Chemistry (INM-5), Wilhelm-Johnen-Str., 52428 Jülich, Germany

[2] Institute of Radiochemistry and Experimental Molecular Imaging, Faculty of Medicine and University Hospital Cologne, University of Cologne, Kerpener Str. 62, 50937 Cologne, Germany

* corresponding author: b.neumaier@fz-juelich.de

Table of contents

1. NMR Spectra 2
   1.1. Compound **3** 2
   1.2. Compound **4** 3
   1.3. Compound **5** 4
   1.4. Compound **6** 5
   1.5. Compound **7** 6
   1.6. Compound **8** 7
   1.7. Compound **8a**: 8
   1.8. Compound **9** 9
   1.9. Compound **10** 10
   1.10. Compound **10a** 11
   1.11. Compound **11** 12
   1.12. Compound **12** 13
2. HPLC and GC chromatograms 14

1. NMR Spectra

1.1. Compound **3**

^{1}H-NMR (400 MHz, DMSO-d_6)

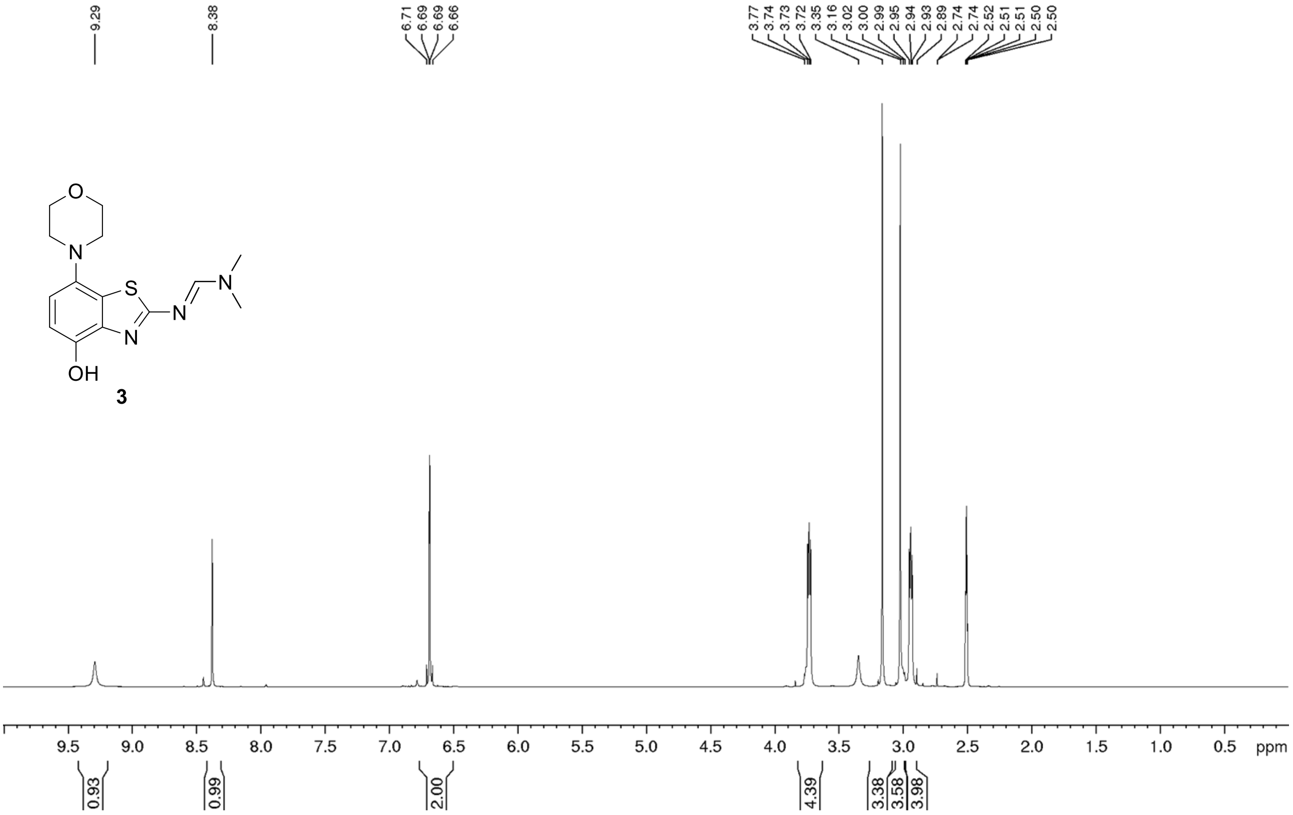

^{13}C-NMR (101 MHz, DMSO-d_6)

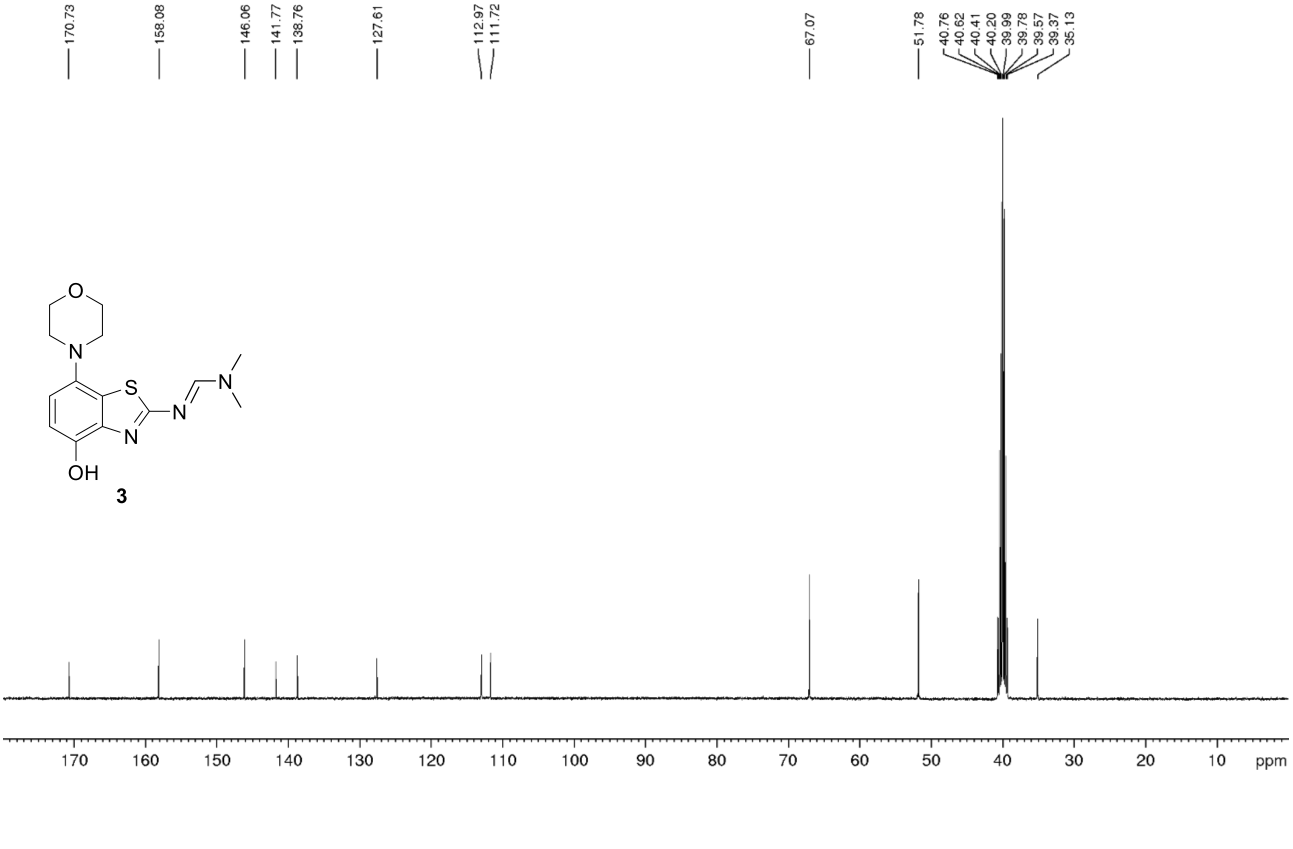

1.2. Compound **4**

^{1}H-NMR (400 MHz, DMSO-d_6)

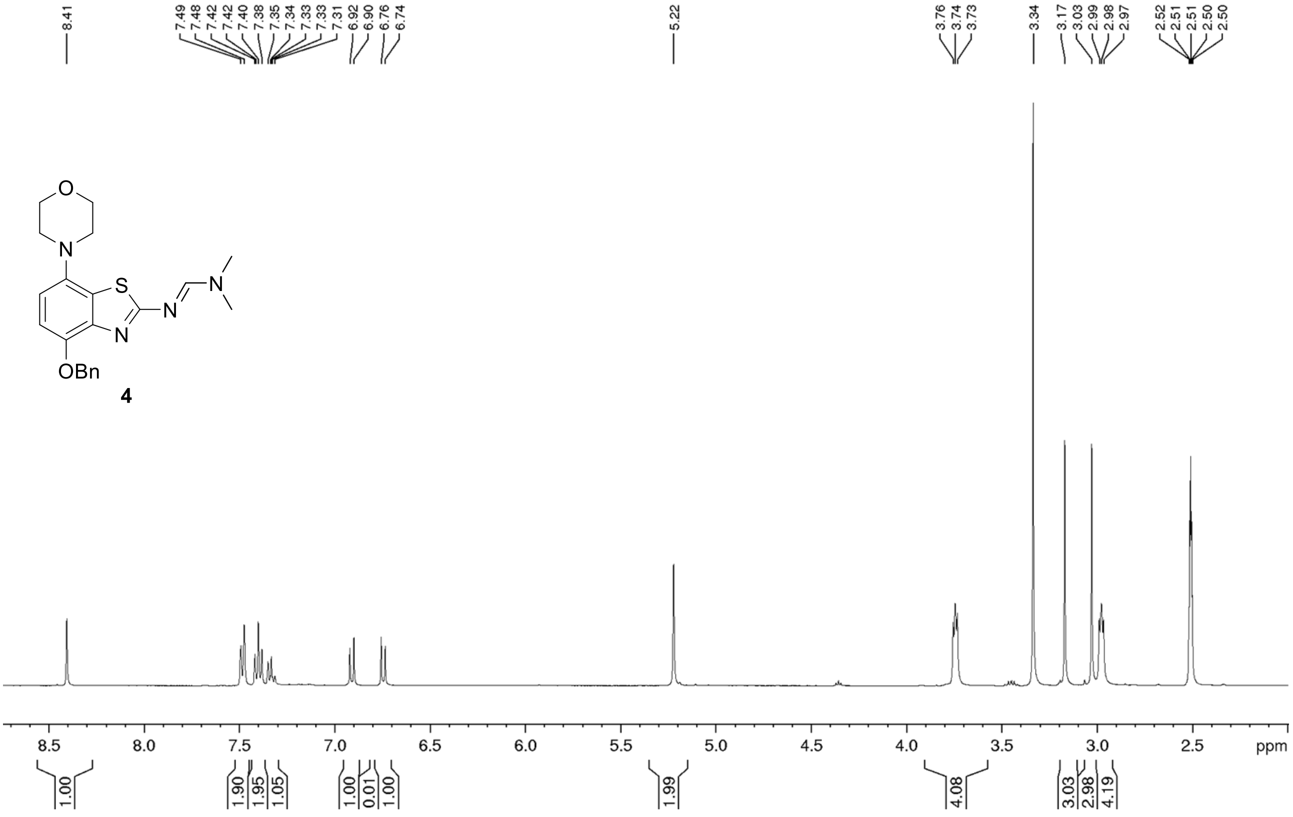

^{13}C-NMR (101 MHz, DMSO-d_6)

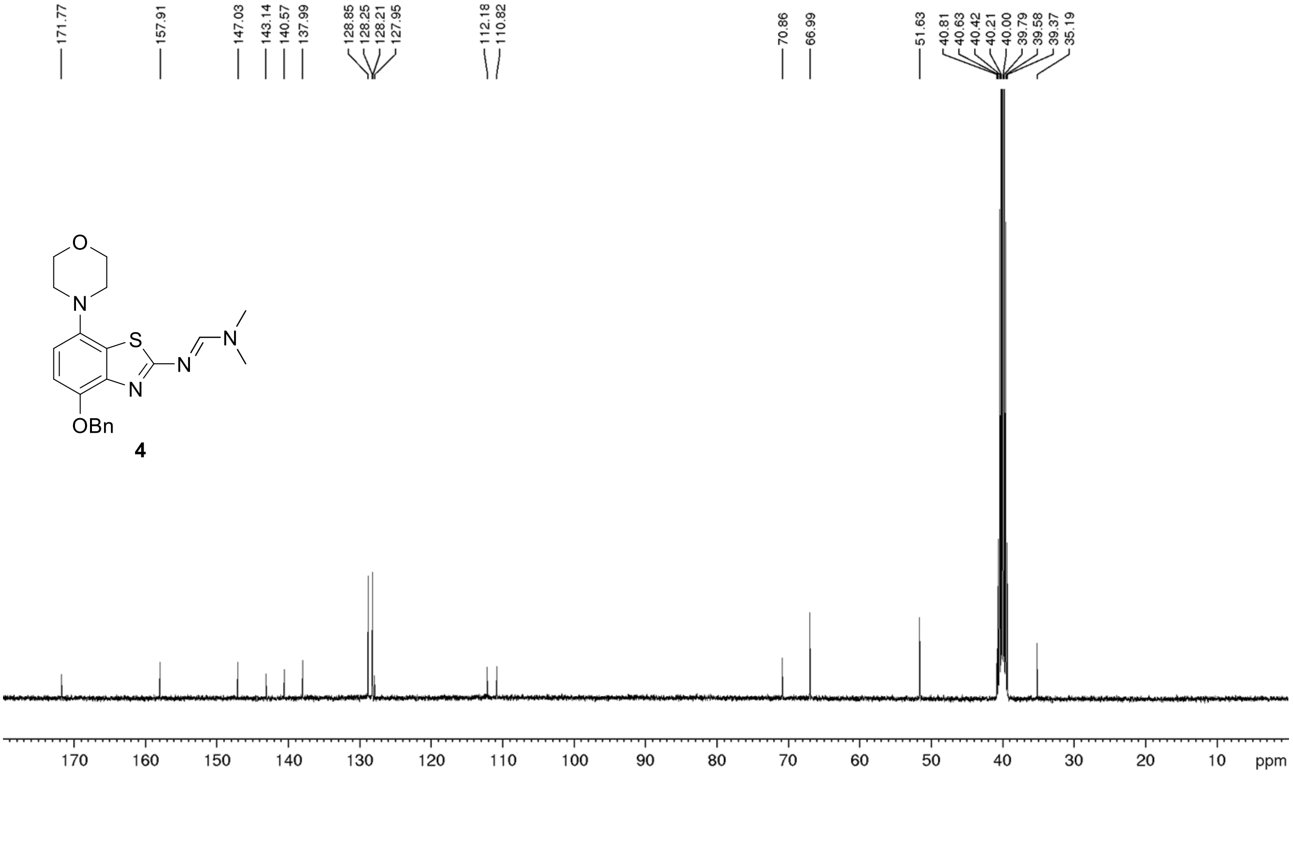

1.3. Compound **5**

^{1}H-NMR (400 MHz, DMSO-d_6)

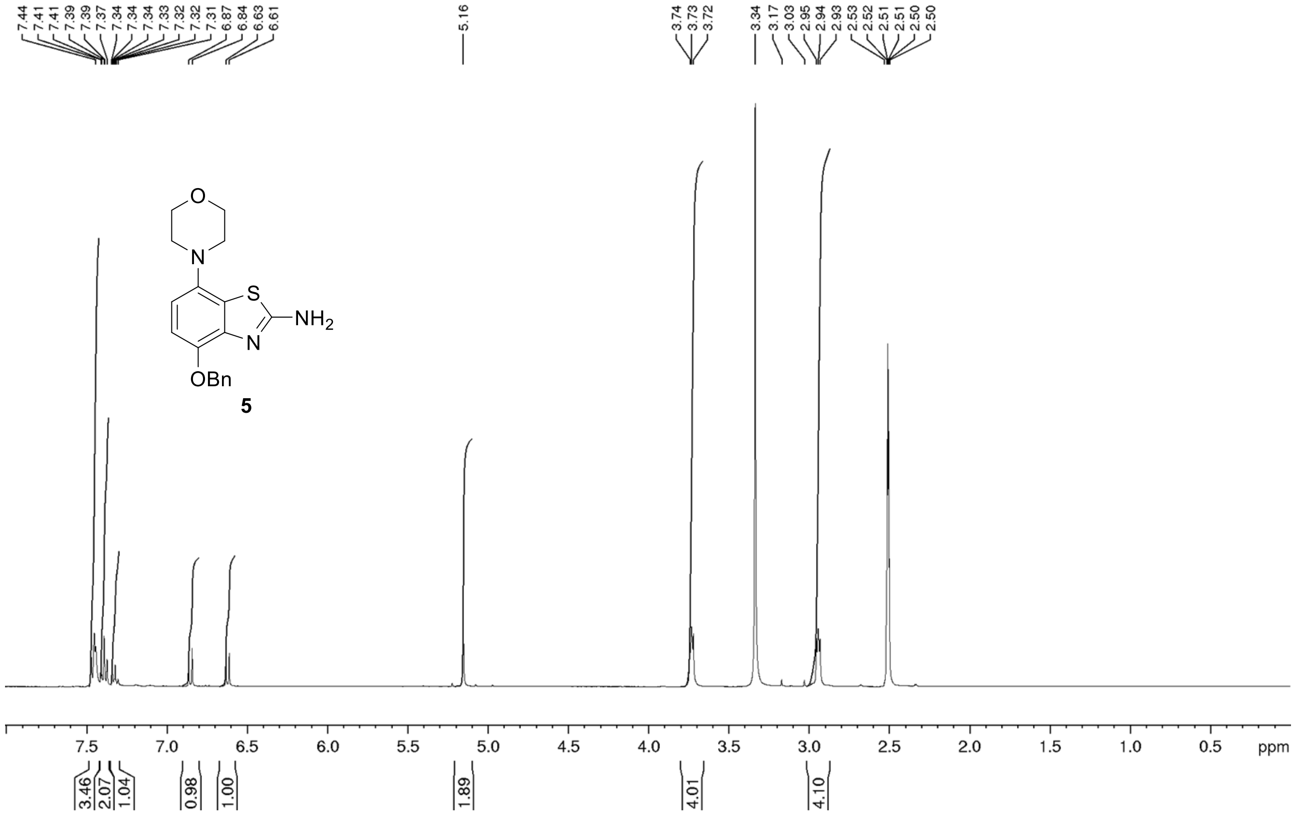

^{13}C-NMR (101 MHz, DMSO-d_6)

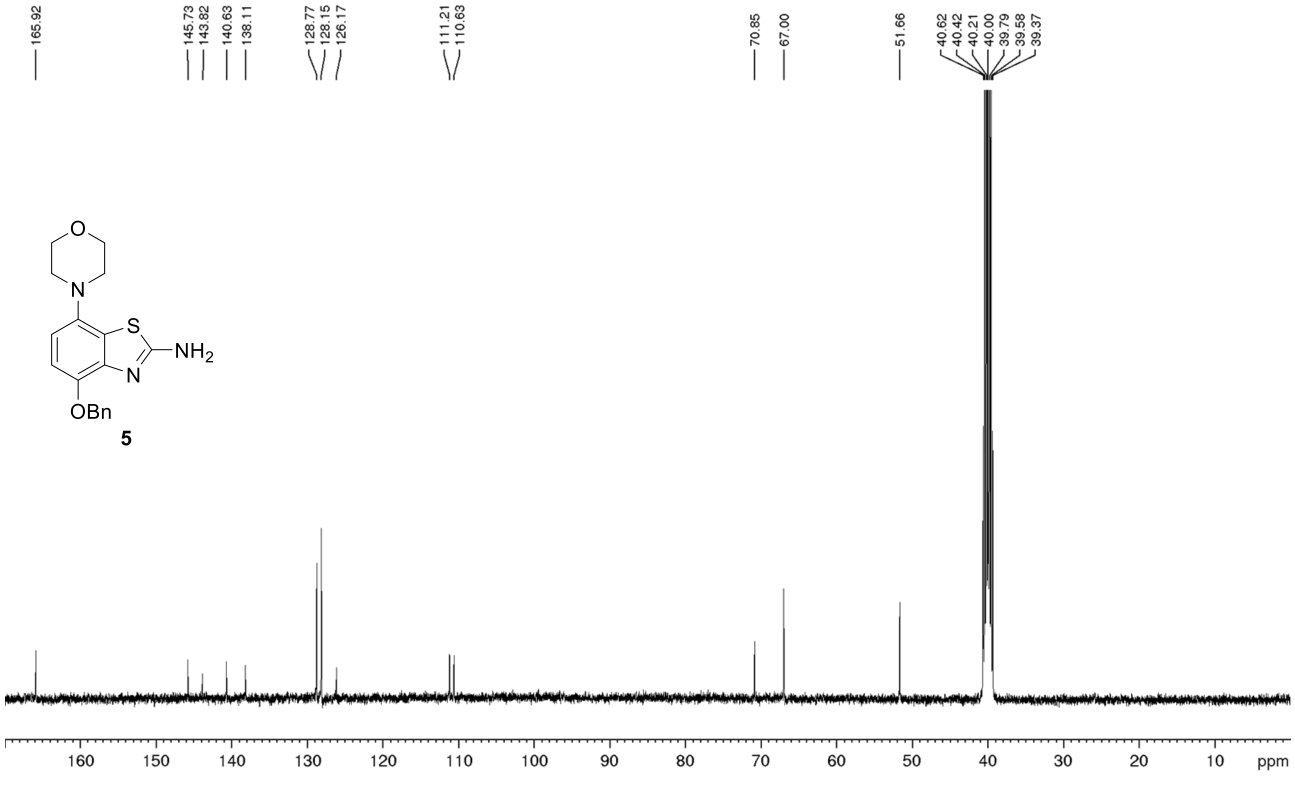

1.4. Compound **6**

^{1}H-NMR (400 MHz, DMSO-d_6)

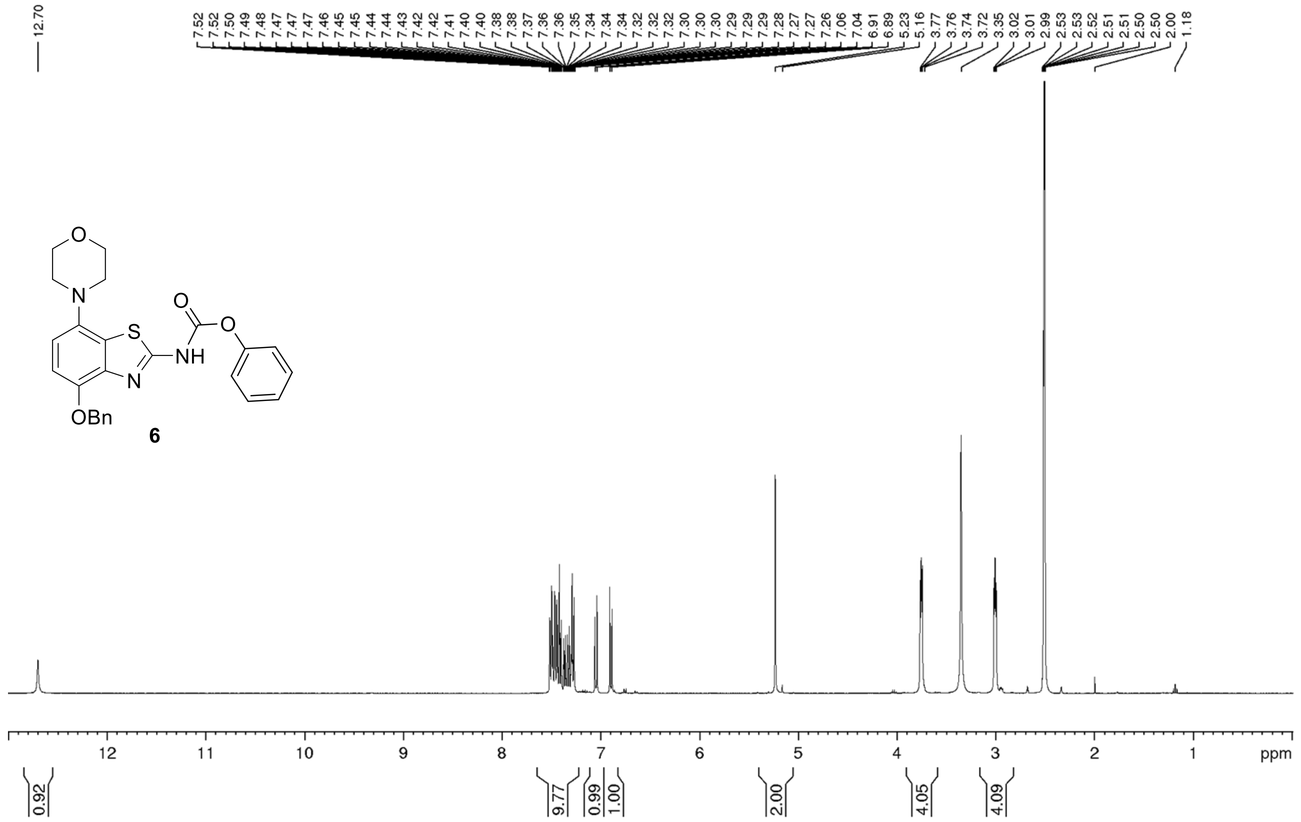

^{13}C-NMR (101 MHz, DMSO-d_6)

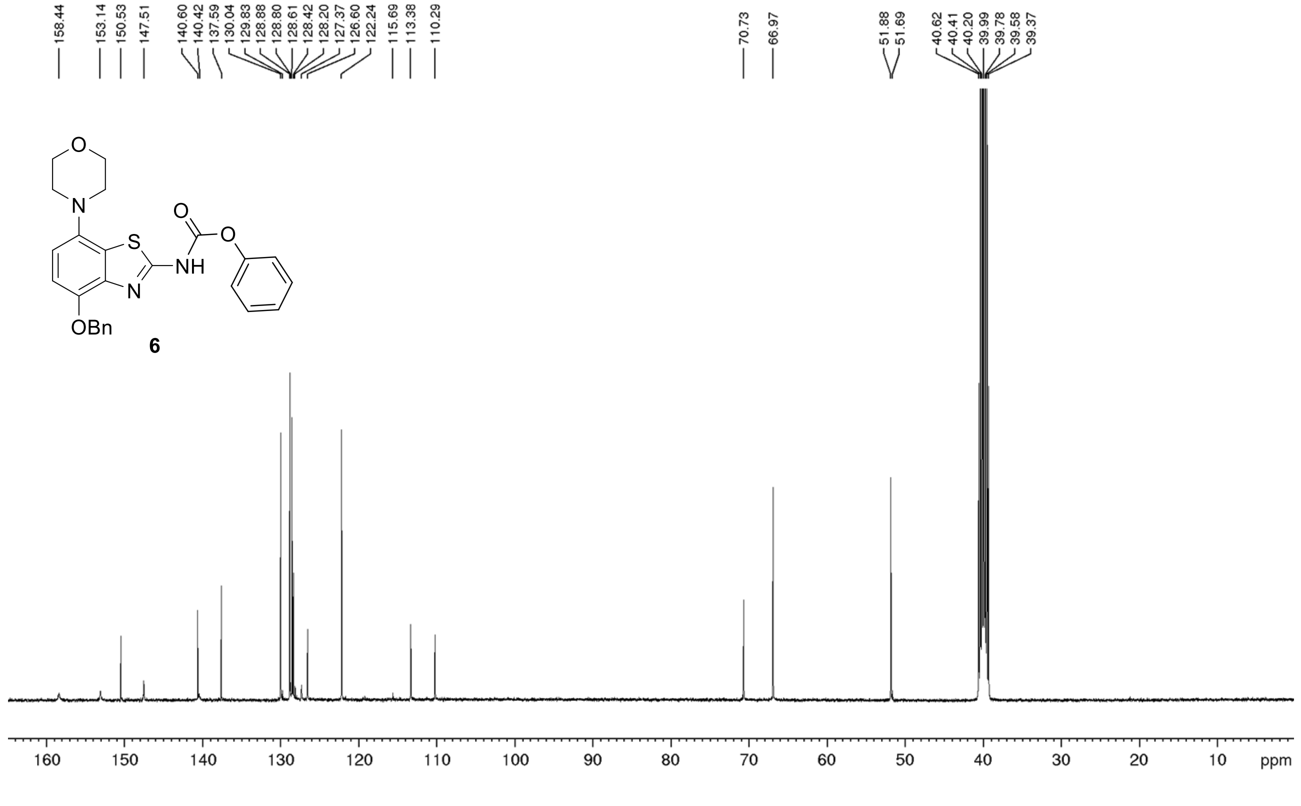

1.5. Compound **7**

^{1}H-NMR (400 MHz, DMSO-d_6)

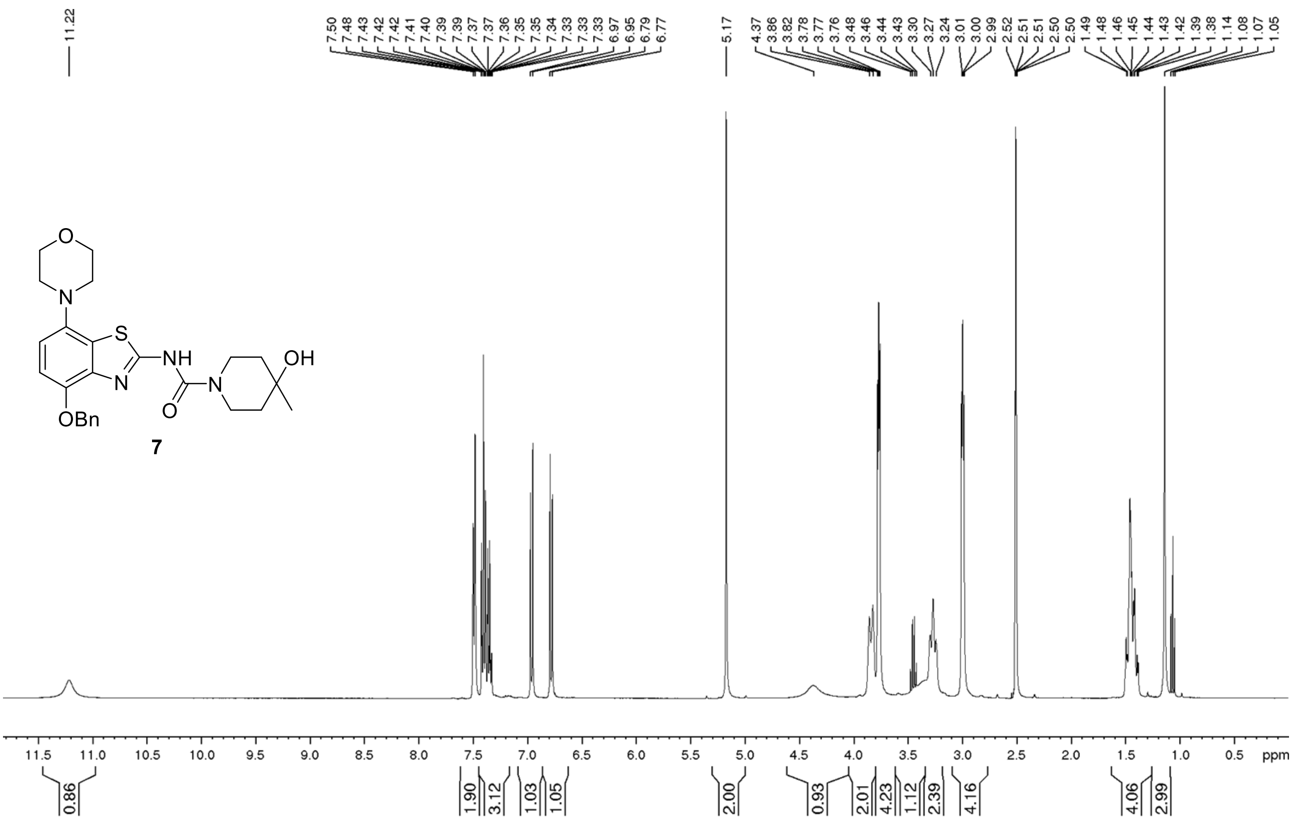

^{13}C-NMR (101 MHz, DMSO-d_6)

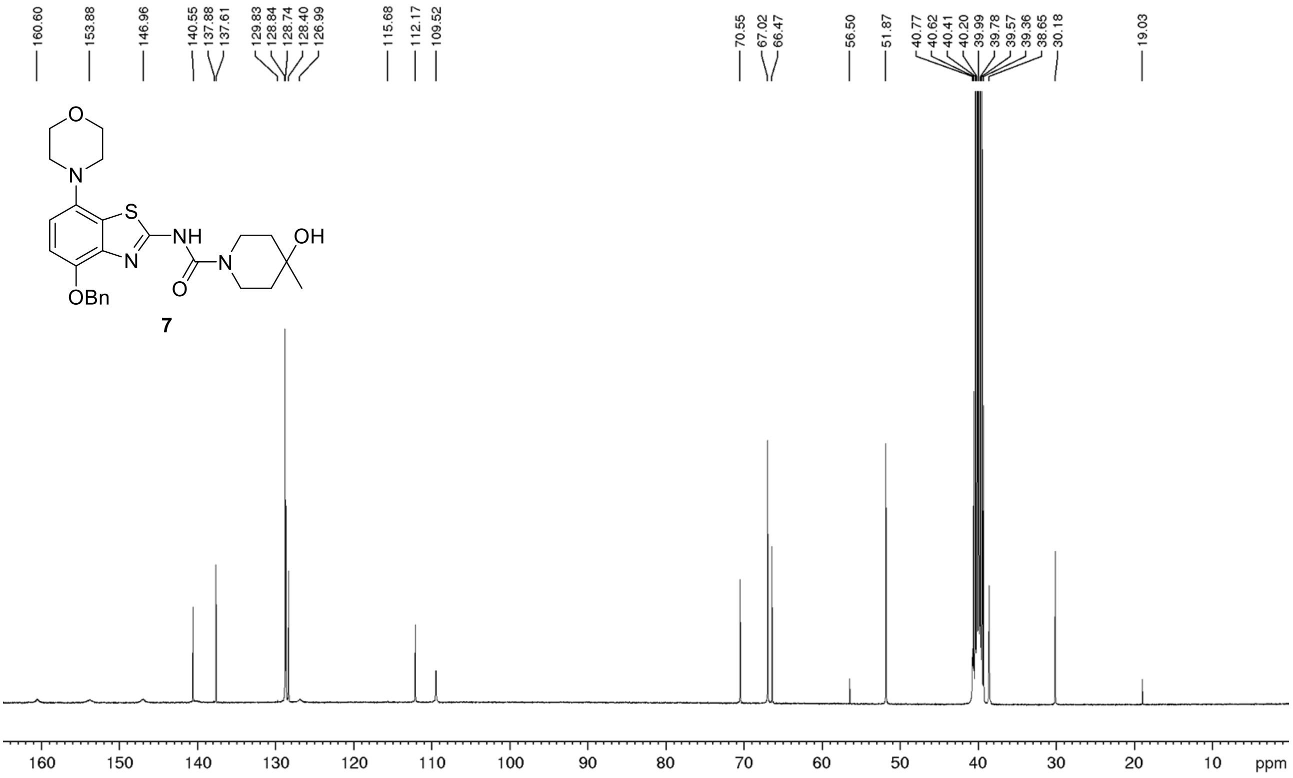

1.6. Compound **8**

^{1}H-NMR (400 MHz, DMSO-d_6)

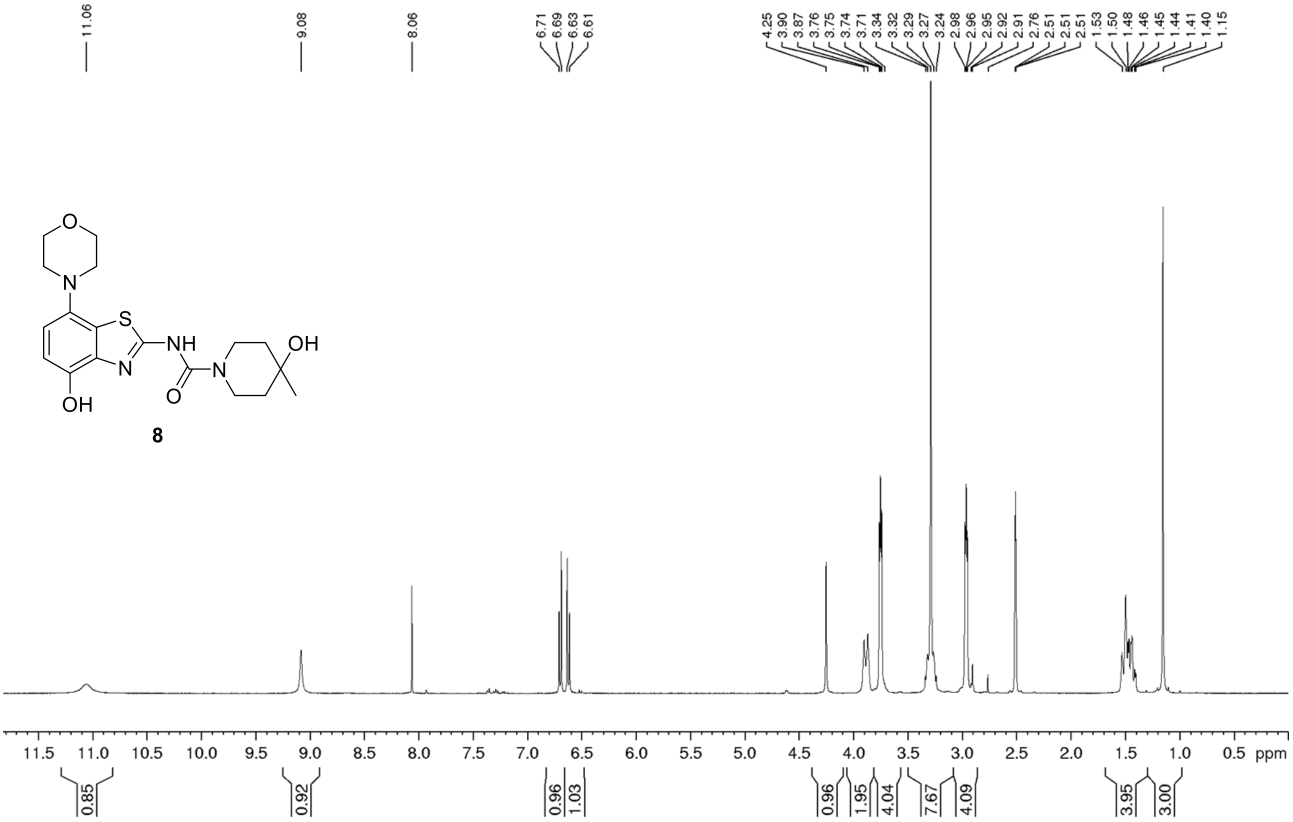

^{13}C-NMR (101 MHz, DMSO-d_6)

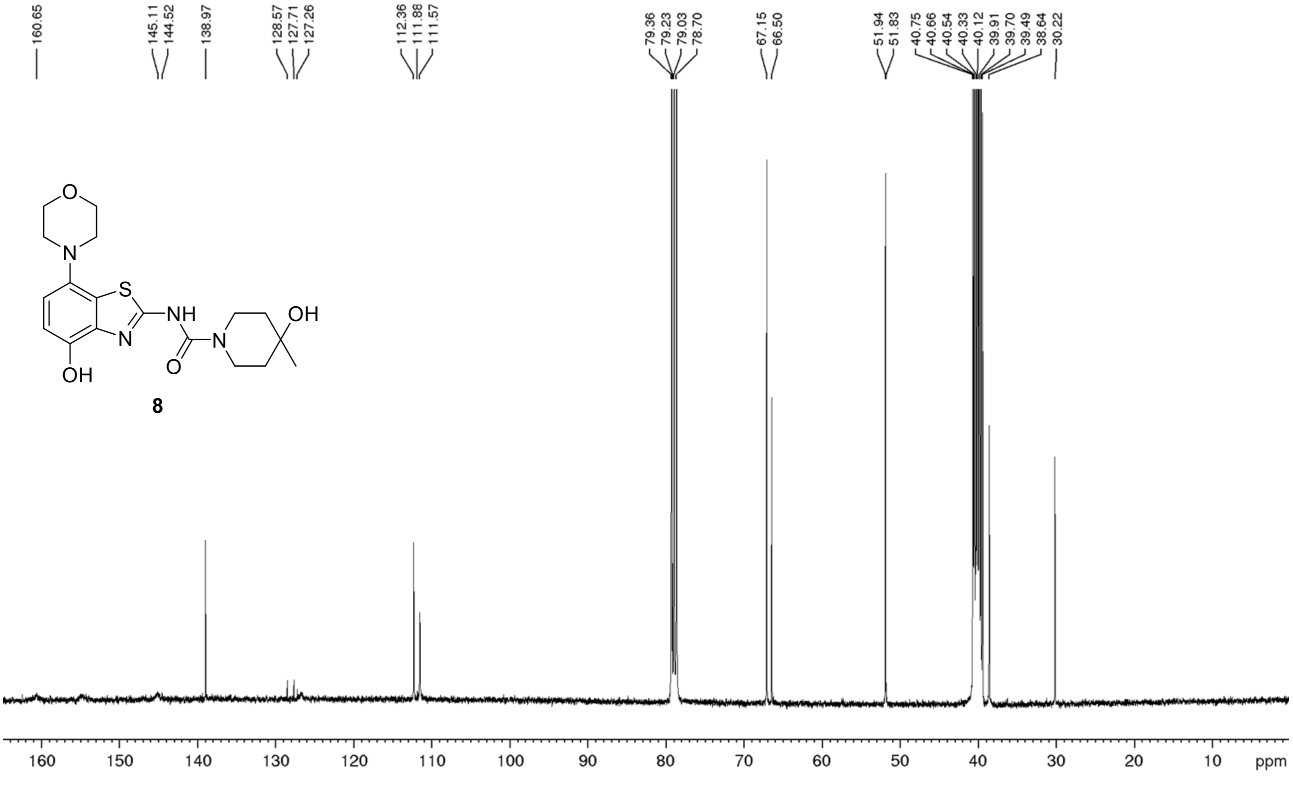

1.7. Compound **8a**:

^{1}H-NMR (400 MHz, $CDCl_3$)

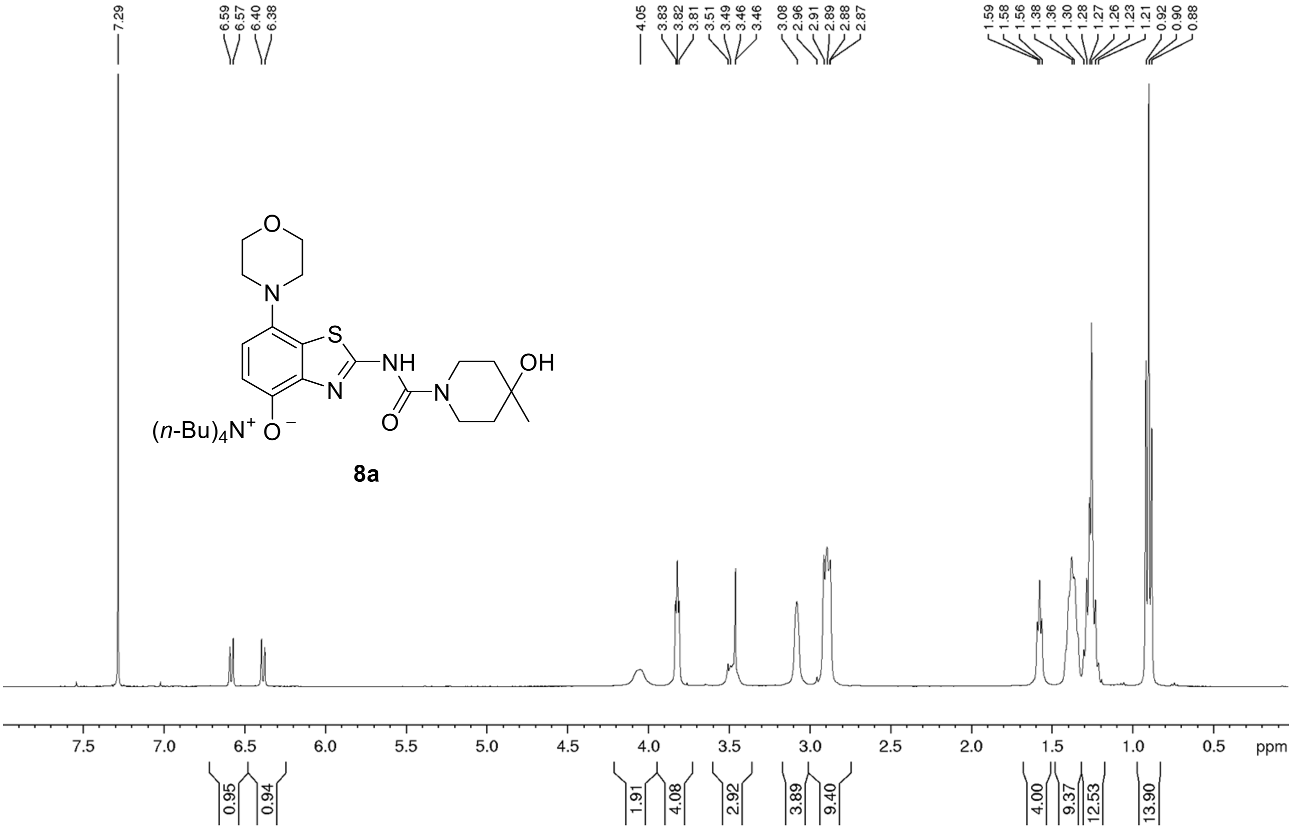

^{13}C-NMR (101 MHz, $CDCl_3$)

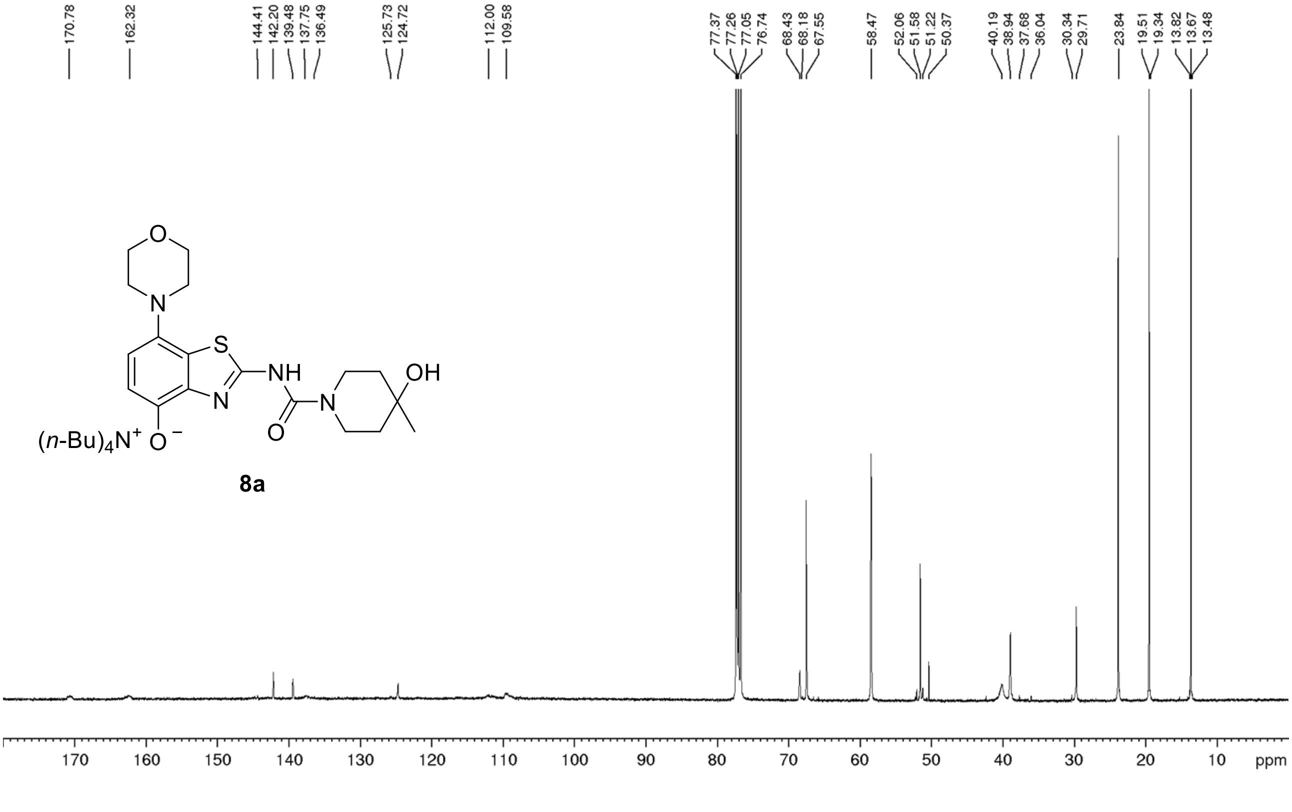

1.8. Compound **9**

^{1}H-NMR (400 MHz, DMSO-d_6)

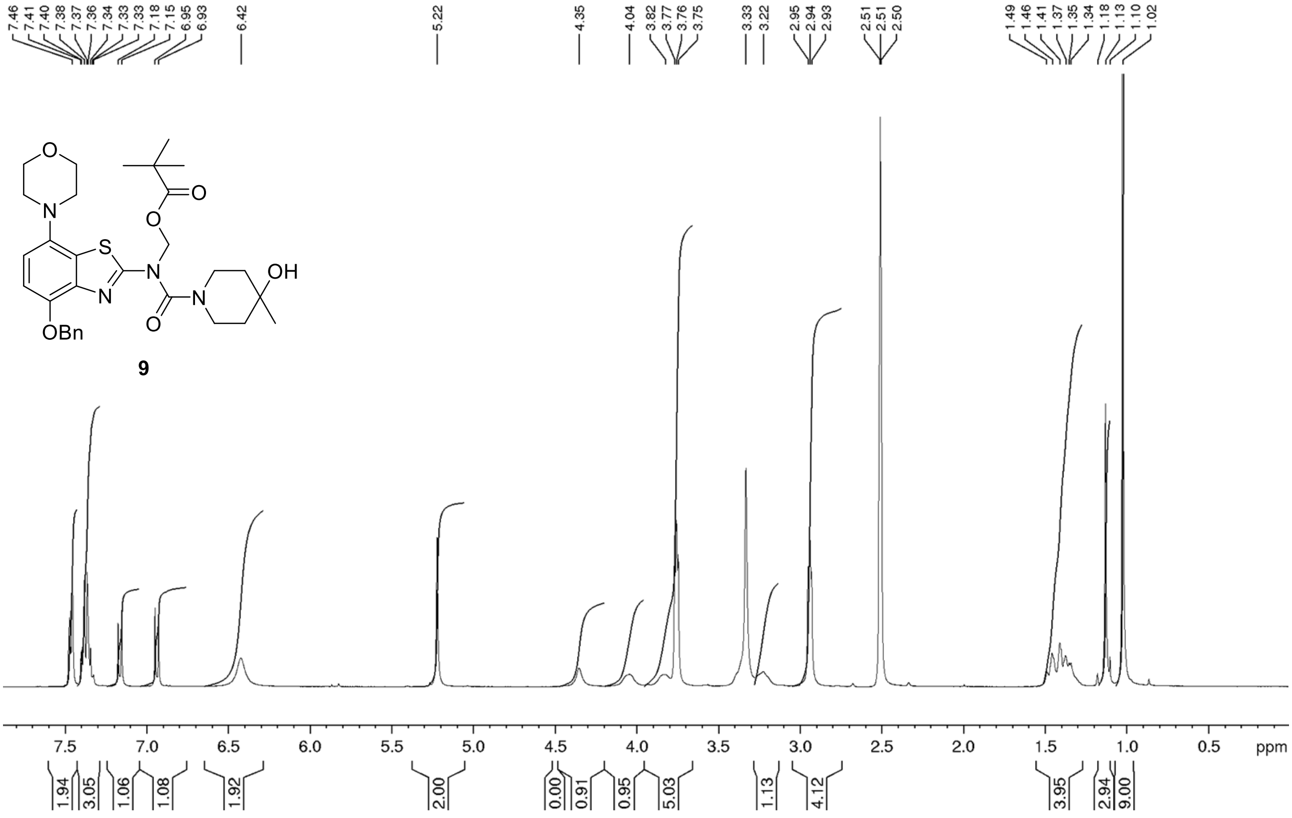

^{13}C-NMR (101 MHz, DMSO-d_6)

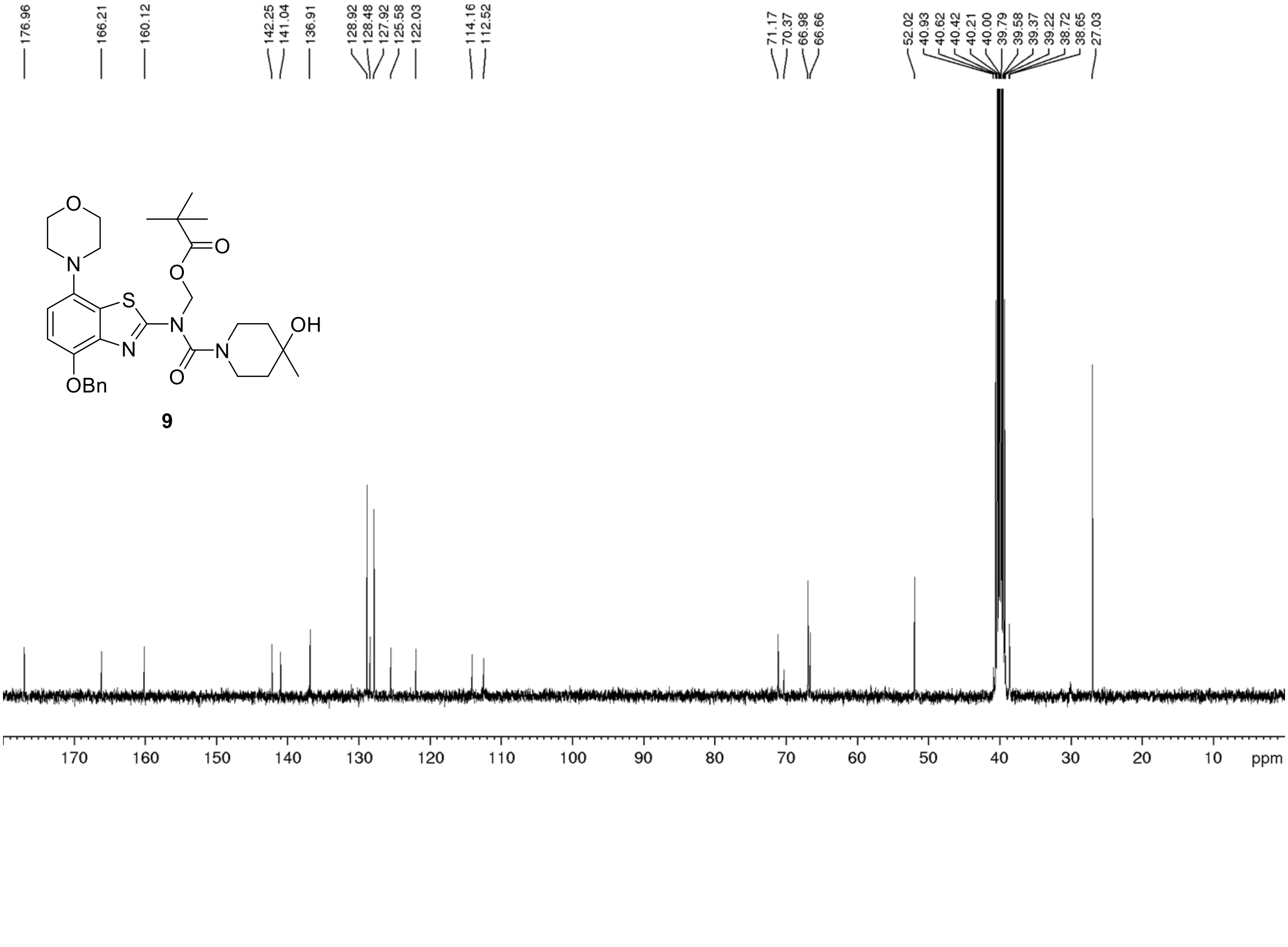

1.9. Compound **10**

^{1}H-NMR (400 MHz, DMSO-d_6)

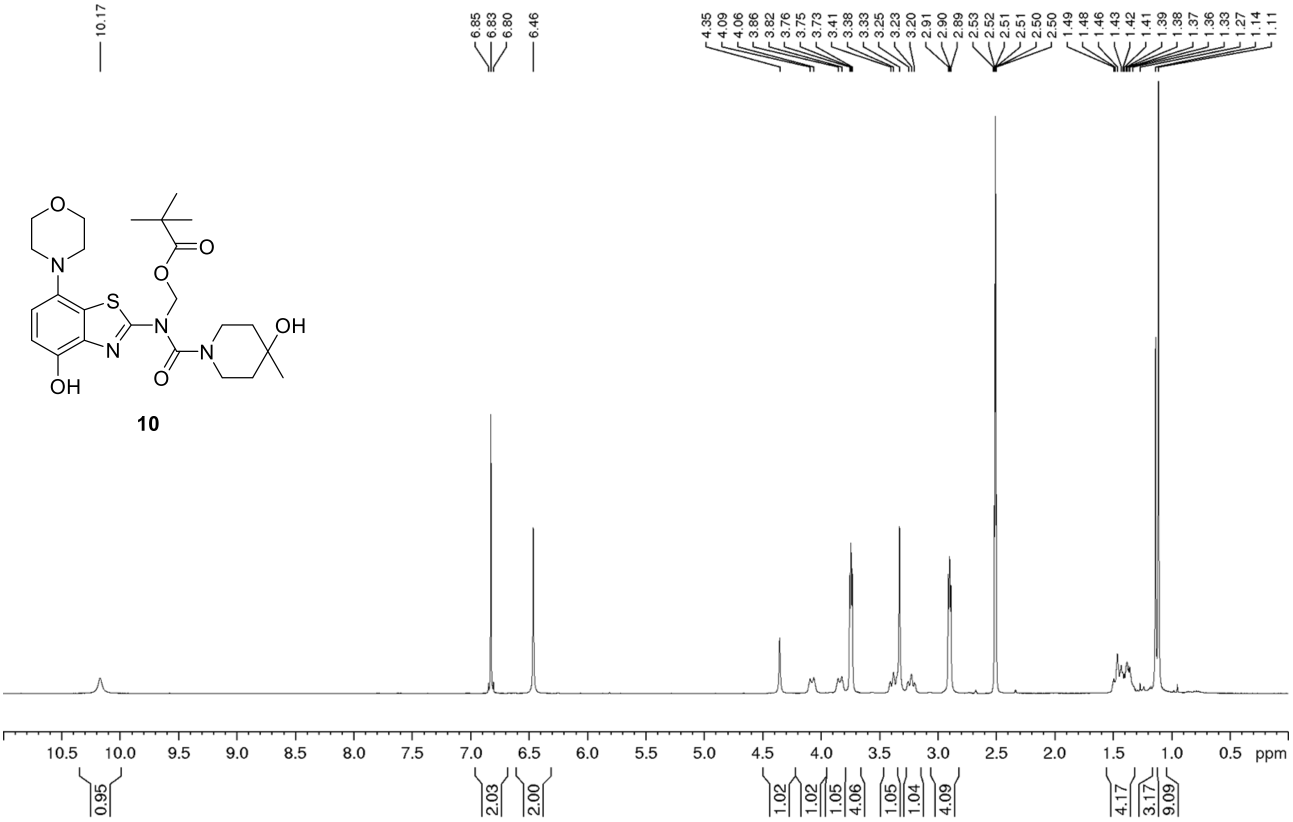

^{13}C-NMR (101 MHz, DMSO-d_6)

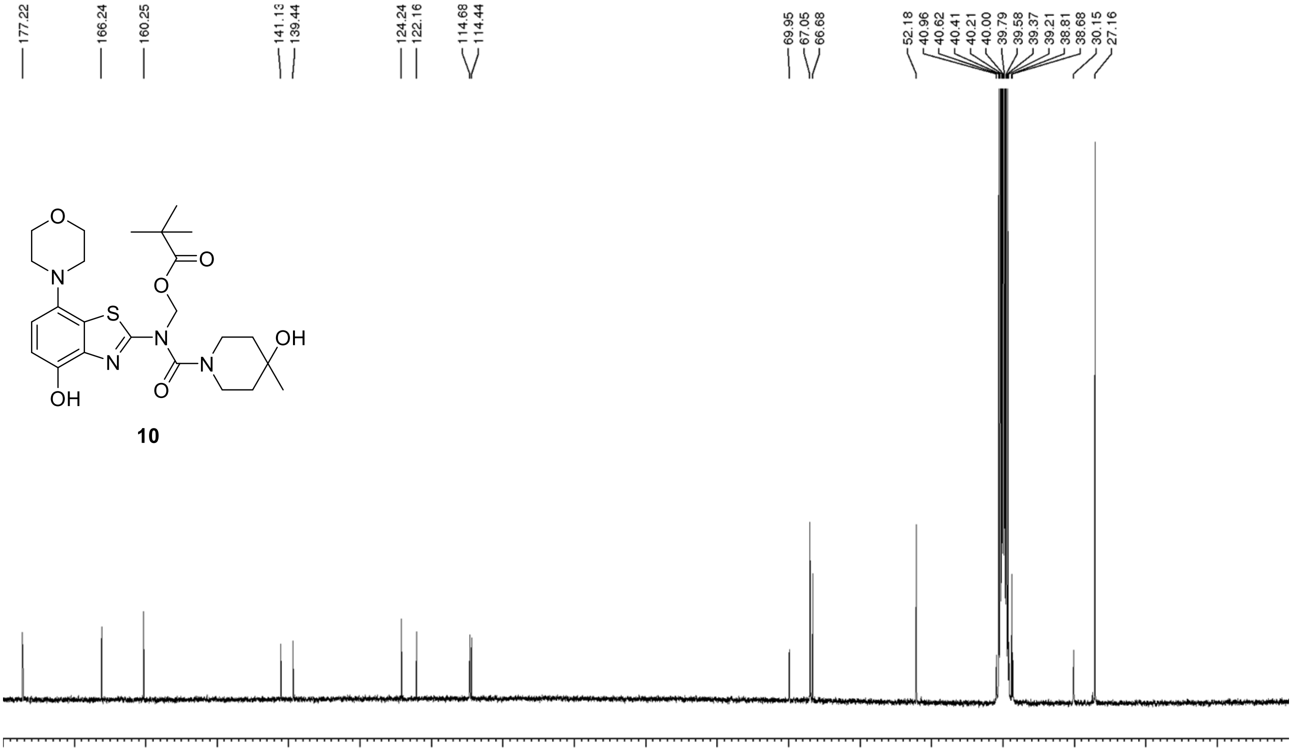

1.10. Compound **10a**

<u>^{1}H-NMR (400 MHz, $CDCl_3$)</u>

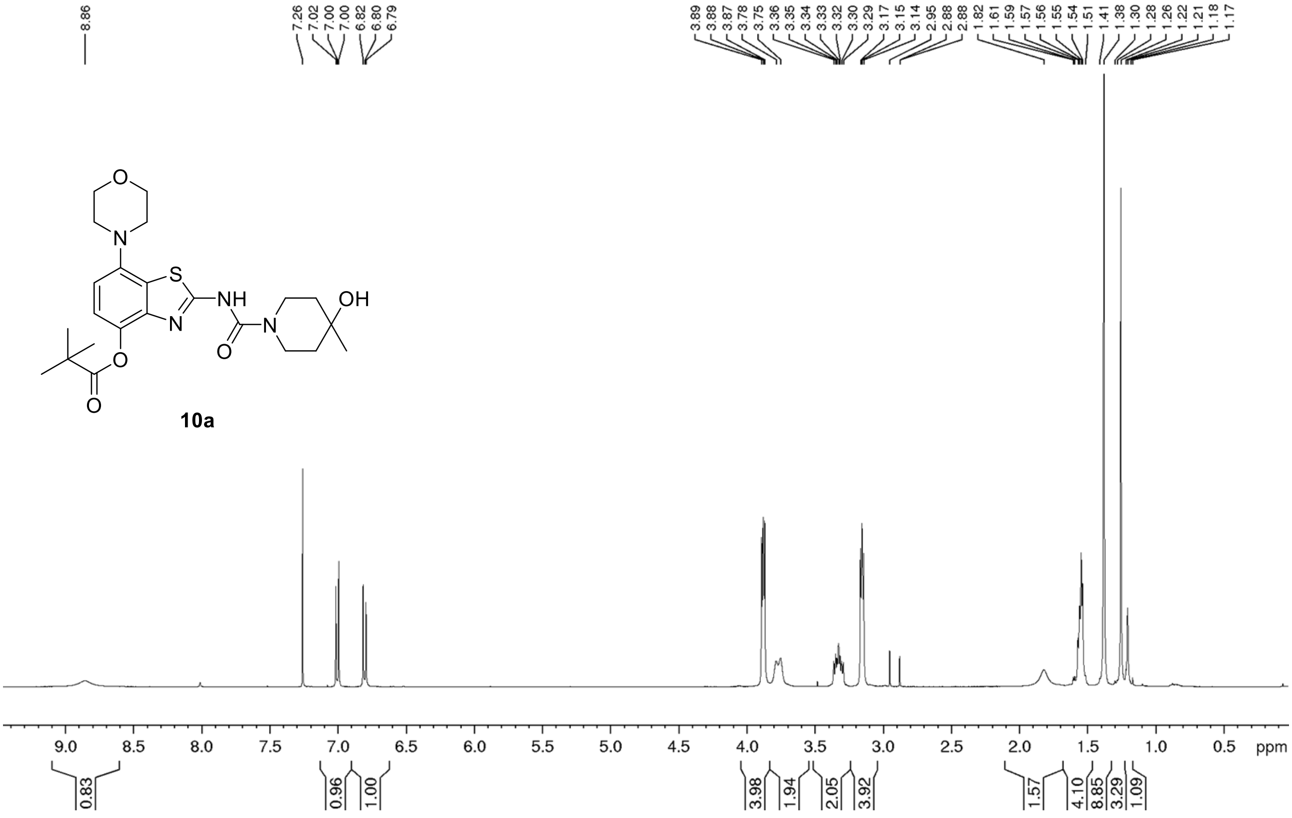

<u>^{13}C-NMR (101 MHz, $CDCl_3$)</u>

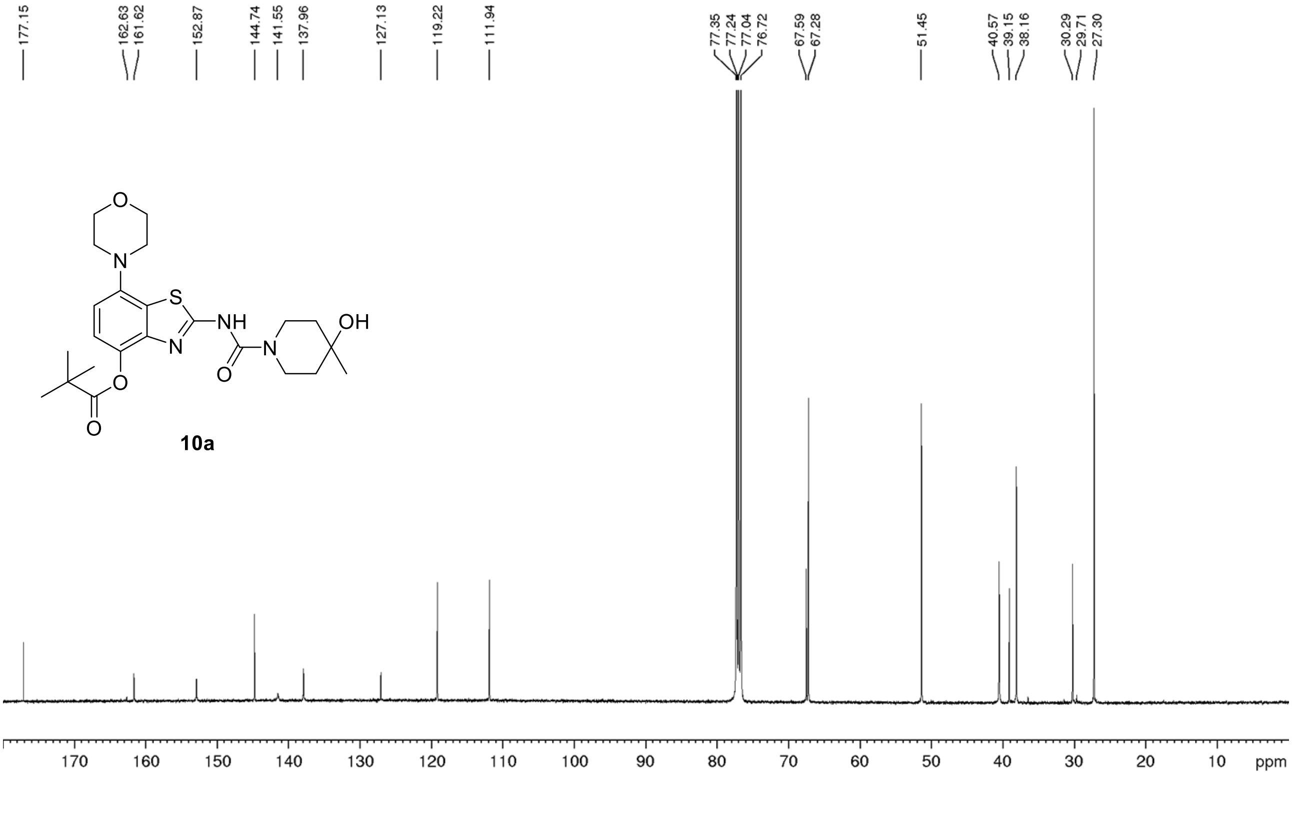

1.11. Compound **11**

^{1}H-NMR (400 MHz, DMSO-d_6)

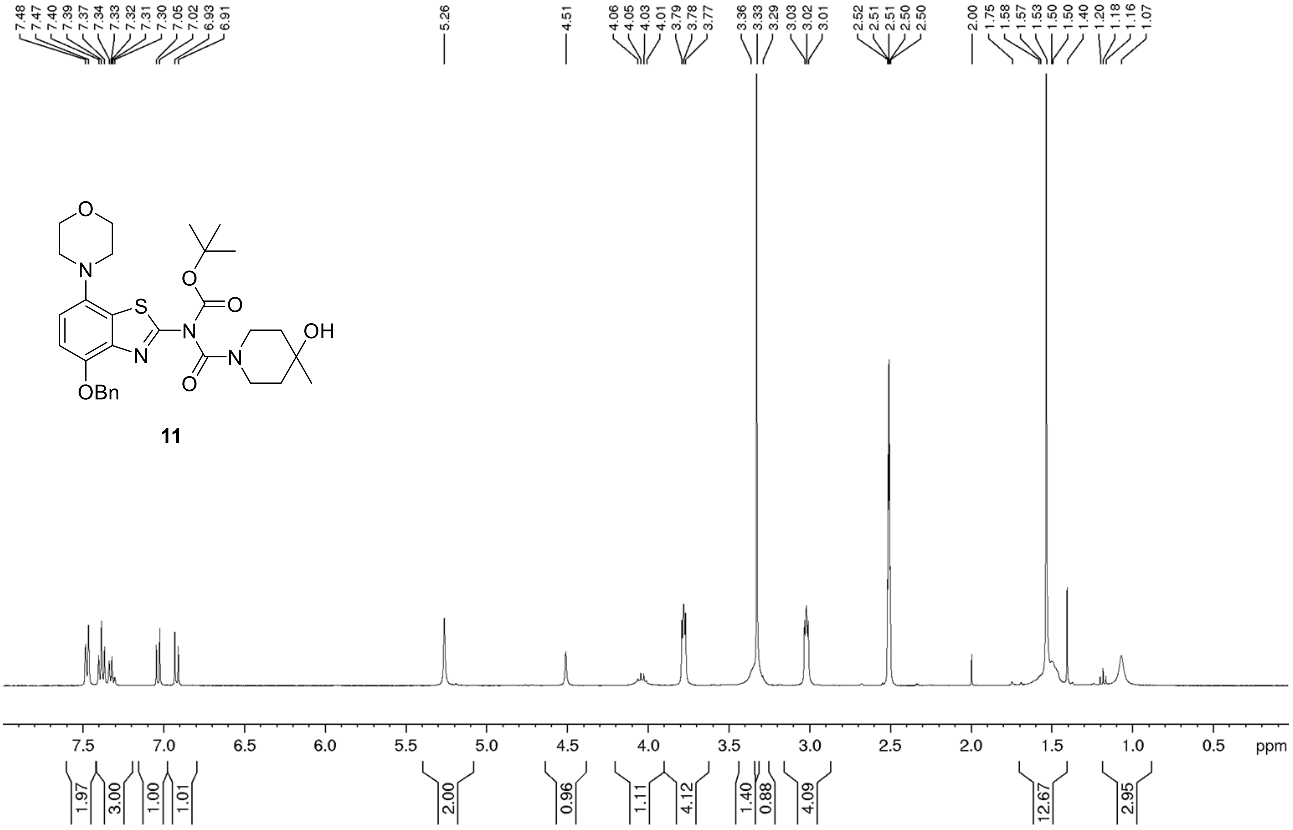

^{13}C-NMR (DEPT135Q, 101 MHz, DMSO-d_6)

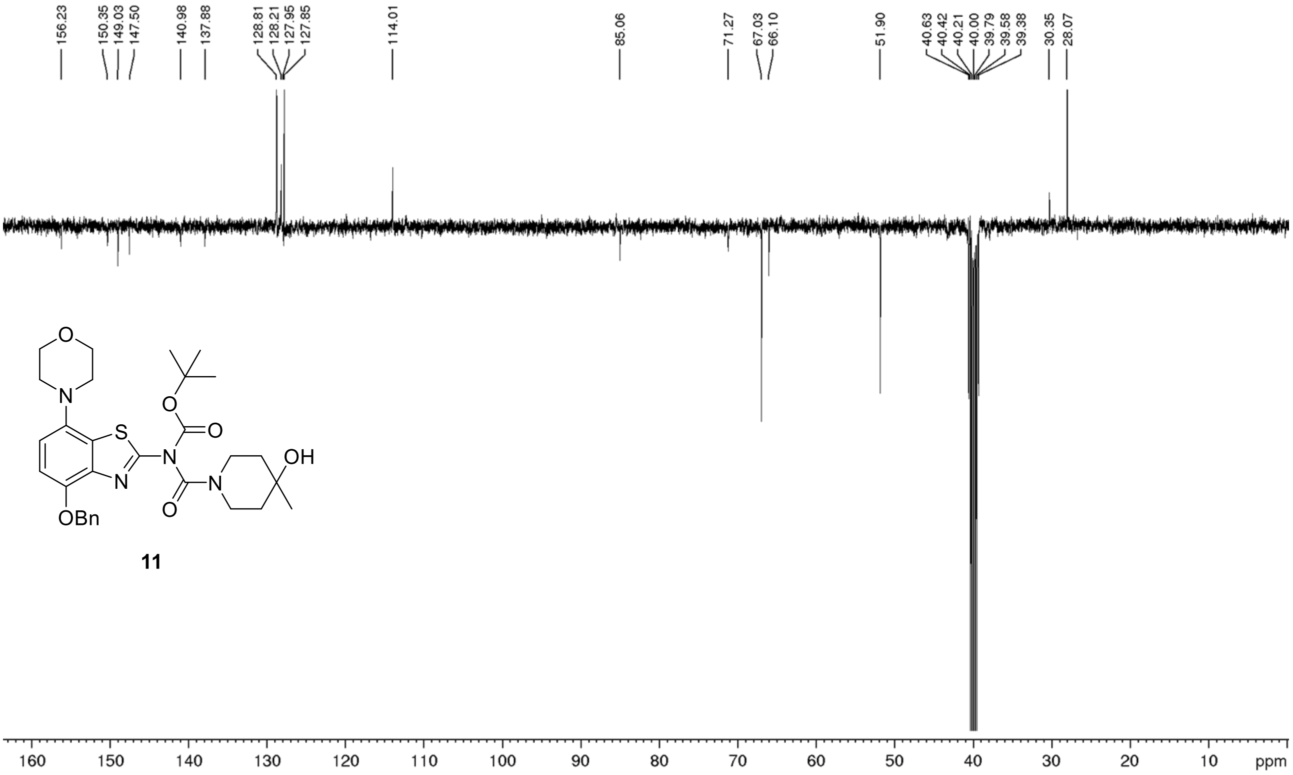

1.12. Compound **12**

<ins>^{1}H-NMR (400 MHz, DMSO-d_6)</ins>

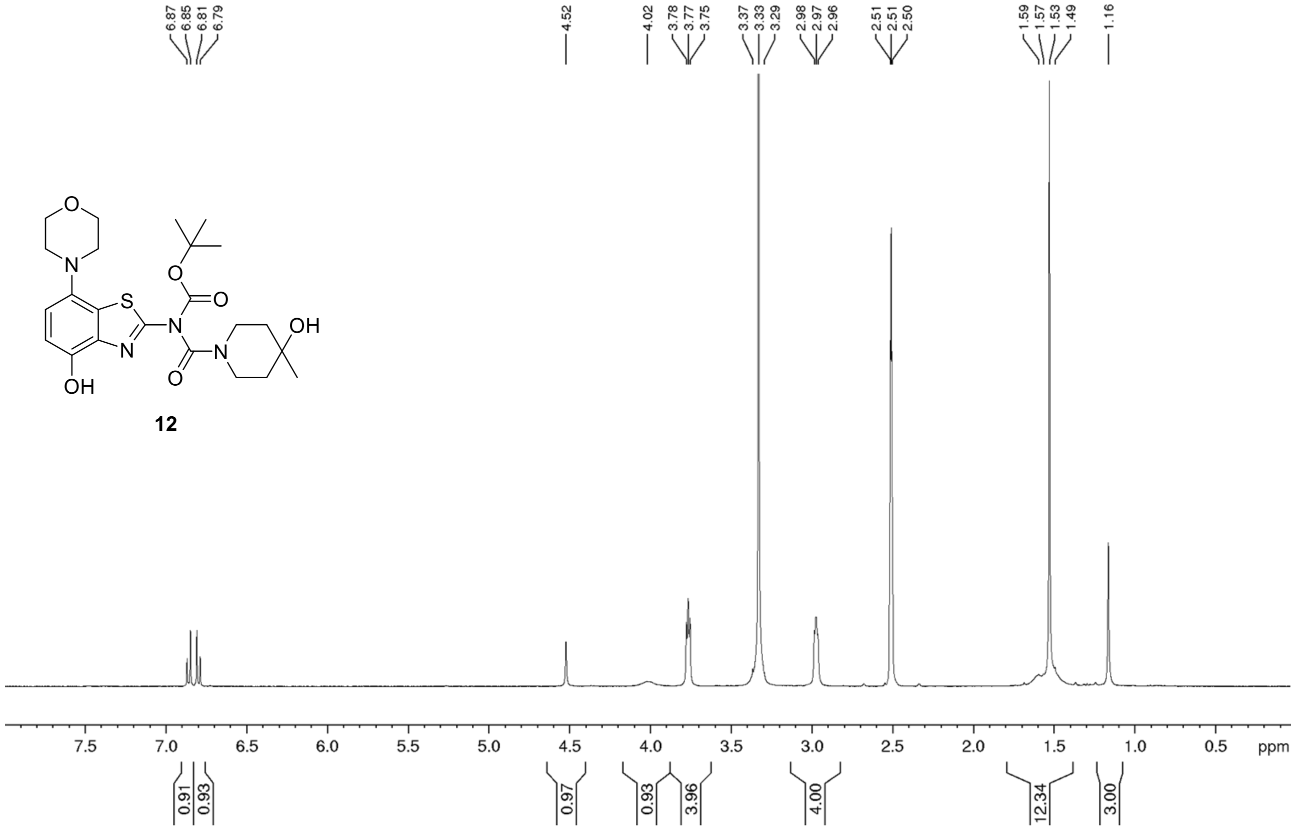

<ins>^{13}C-NMR (101 MHz, DMSO-d_6)</ins>

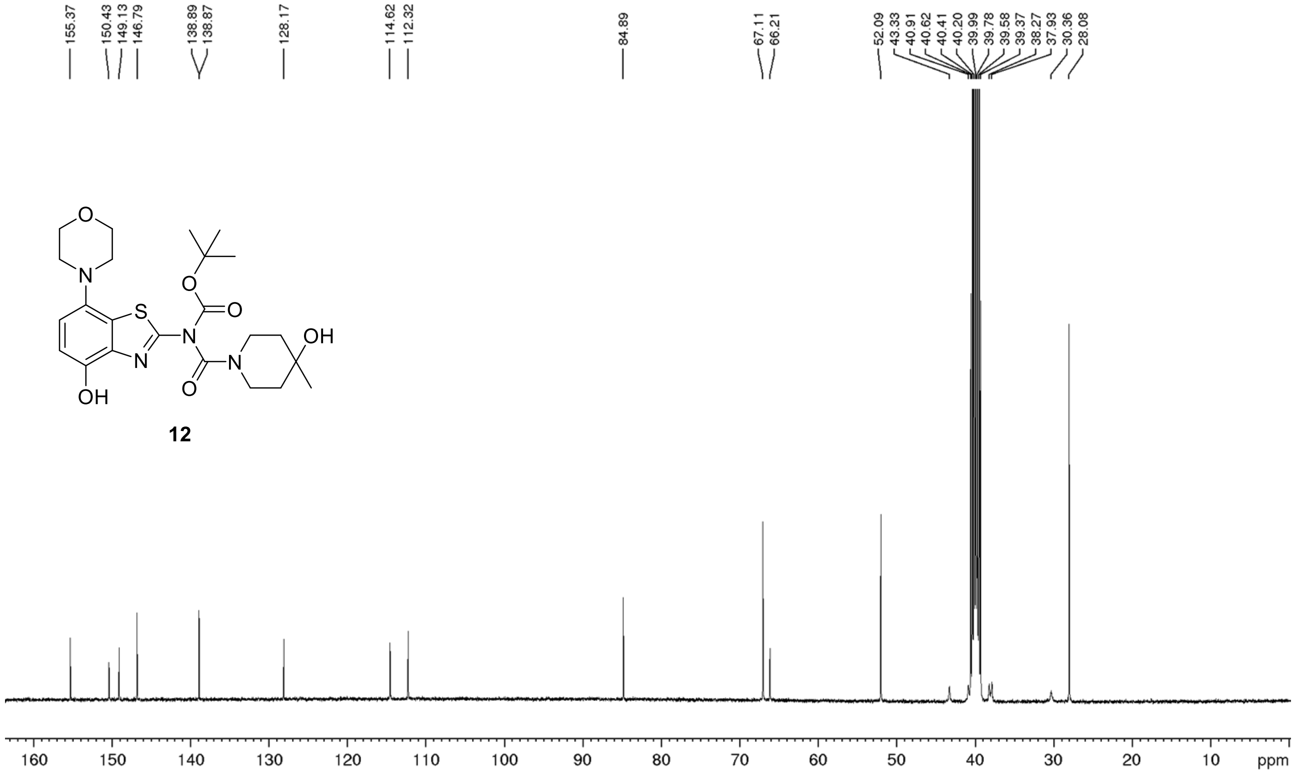

2. HPLC and GC chromatograms

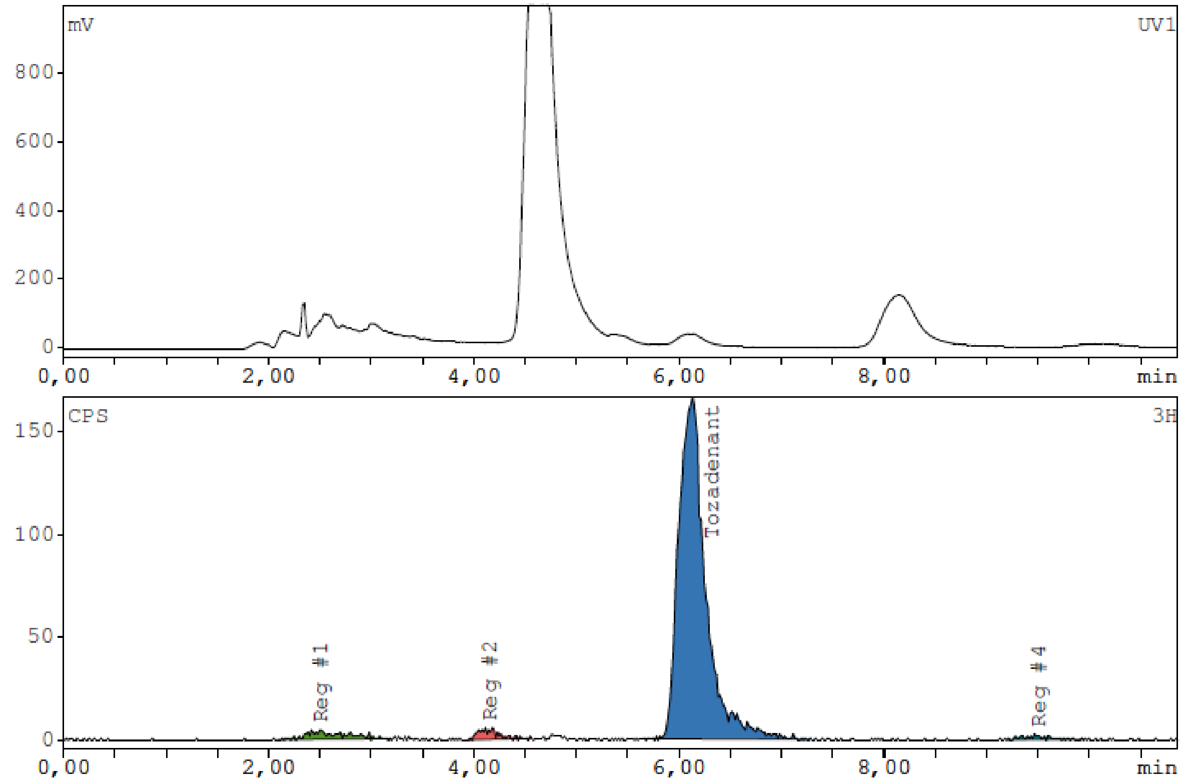

Figure S1: HPLC chromatogram of the semipreparative purification of [^{11}C]tozadenant (top: UV trace at 254 nm, bottom: radiotrace). HPLC conditions: Column: Kromasil® ace-EPS 250 × 8 mm; eluent: 60% $MeOH_{aq}$; flow-rate: 4.0 mL/min.

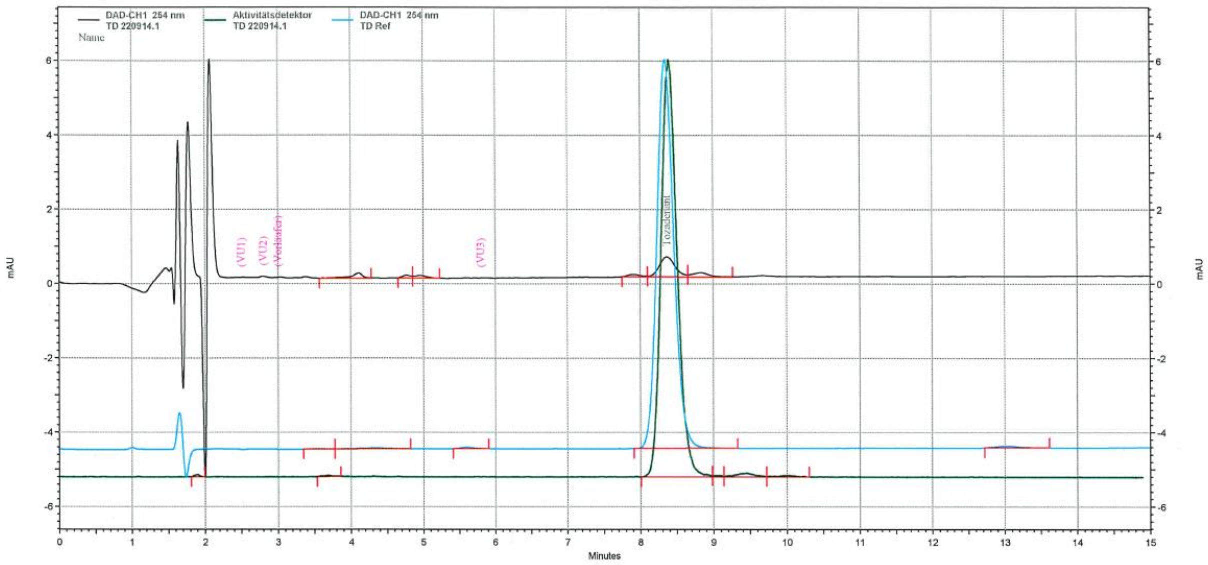

Figure S2: Overlay of the HPLC chromatograms of purified and formulated [^{11}C]tozadenant (black: UV trace at 254 nm, green: radiotrace) and the non-radioactive reference compound (blue: UV trace at 254 nm). HPLC conditions: Column: Kromasil® C18 250 × 4.6 mm; eluent: 60% $MeOH_{aq}$; flow rate: 1.0 mL/min.

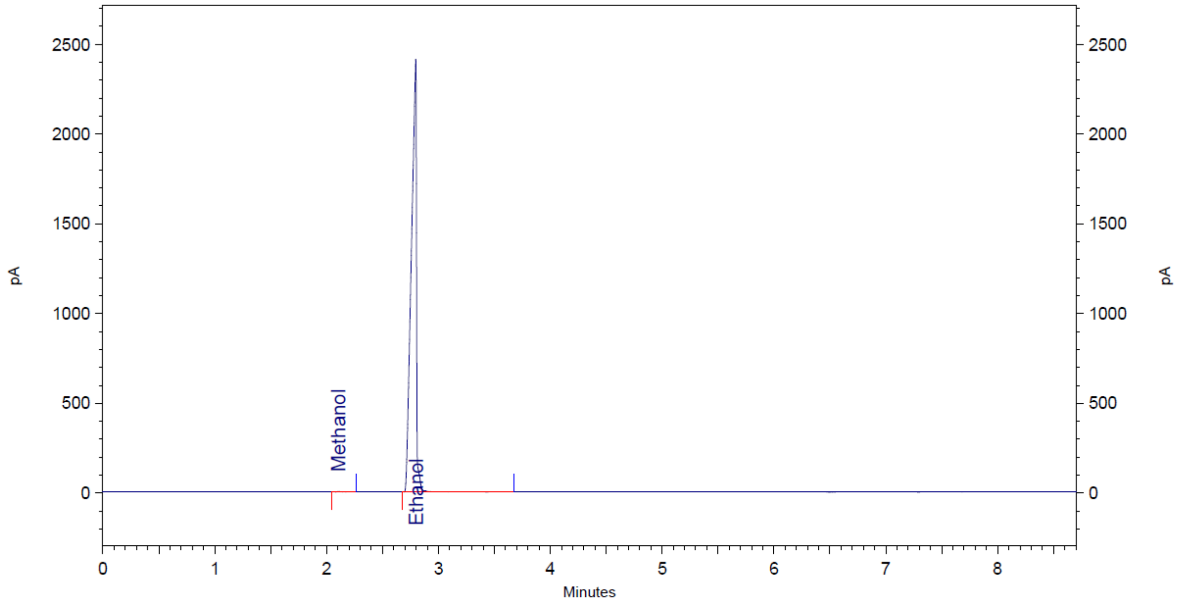

Figure S3: GC chromatogram of purified and formulated [^{11}C]tozadenant for determination of residual solvents.
